# HairSentinel: a time-aware anomaly detection framework for forecasting hairfall trends using temporal fusion transformers

**DOI:** 10.3389/frai.2025.1649740

**Published:** 2025-10-14

**Authors:** A. Anny Leema, T. Saktheshwaran, G. Reena Sri, P. Balakrishnan

**Affiliations:** ^1^Analytics Department, School of Computer Science and Engineering, Vellore Institute of Technology, Vellore, Tamil Nadu, India; ^2^Department of Data Science, School of Computer Science and Engineering, Vellore Institute of Technology, Vellore, Tamil Nadu, India

**Keywords:** hairfall detection, time series analysis, anomaly detection, hormonal imbalance, predictive modeling, health monitoring, nutritional deficiency, scalp health

## Abstract

Hairfall is a primary concern for many individuals worldwide today. Hair strands may fall due to various conditions such as hereditary factors, scalp health issues, nutritional deficiencies, hormonal fluctuations, or irregular sleep cycles. Our study presents a novel approach to detecting hairfall trends over time. While traditional methods infer hairfall rates using CNN and SVM models—classifying types of hairfall based on high-resolution images and complex techniques—this study addresses the issue by analyzing user-provided data through simple, straightforward questions, maintaining ease of use. Each attribute is collected using a time-centric approach on a daily or weekly basis. For time series anomaly detection, we utilize LSTM, Random Forest, and the Temporal Fusion Transformer (TFT) to model hairfall fluctuations and compare them with the ARIMAX model across various metrics to identify the most suitable one. The TFT model is selected as the most suitable, with 97.5% accuracy and 97.4% precision over other models supporting anomaly detection. This allows us to establish normal margins of deviation from typical hair shedding cycles. This study enables the proactive detection of anomalies, indicating sudden increases or decreases in hairfall due to hormonal fluctuations. The results support the early identification of potential health risks before they become intensified and help suggest appropriate dietary plans.

## 1 Introduction

Hairfall has revolutionized both the medical and business worlds. According to our survey, over 80% of people experience hair loss, highlighting the growing importance of early detection of critical health conditions. As individuals become more cautious about their hairfall rates, they tend to invest more in products to handle the issue. This increasing concern has given rise to business trends, including hair treatments, hair care solutions, and diagnostic services. While these solutions may offer temporary relief, they often fail to detect the underlying severity of the problem or the need to investigate potential health conditions. Existing detection methods typically rely on analyzing images of hairfall and comparing them with early-stage cases to assess risk levels. These methods employ techniques such as CNN, SVM, KNN, and other traditional algorithms to detect hairfall. In contrast, our research adopts a scientific and data-driven approach, where data is collected using time as the primary factor and tracked on a daily or weekly basis, which utilizes large volumes of numerical data processed through successive encodings. This method eliminates the dependency on high-resolution images, which often require powerful workstations for large-scale computation.

Studies are conducted to identify key contributing factors that influence hairfall rates, aiming to enhance prediction and accuracy. Attributes such as biological factors, environmental influences, hereditary conditions, hair-related factors, and lifestyle patterns are found to have a meaningful impact on hairfall trends over time. The selection of these features was based on insights from several studies and validated through performance-based feature extraction methods. Unlike traditional methods, our approach detects anomalies and embarks on a time-centric pattern that highlights irregularities and fluctuations in hairfall over specific periods. These spikes act as early indicators of potential health threats, enabling timely intervention and promoting both healthcare awareness and personal care through appropriate dietary adjustments.

The main purpose of the research specifically deals with machine learning-based techniques that include LSTM, Random Forest, and the Temporal Fusion Transformer (TFT), compared to the ARIMAX model, which is a variant of the ARIMA model. The proposed detective model is among those tested and compared, using various statistical measures to evaluate the R-squared score, mean square error, accuracy, specificity, sensitivity, precision, and recall. The adoption of machine learning algorithms has opened up the possibility of equipping each individual with the power of self-health monitoring, especially for hair health and overall wellbeing.

## 2 Literature survey

Recent advancements in artificial intelligence (AI) and deep learning have led to innovative methods for detecting hair loss and scalp-related issues. These new methods employ techniques such as grouping similar data (clustering), identifying patterns in images (image classification), predicting future events (time series forecasting), and combining multiple models (hybrid ensemble models). This section of the literature review explains how different studies have contributed to this area of research.

### 2.1 AI and ML-based hair fall prediction

For example, Farooq et al. (2024) developed an AI-based model to predict hair fall patterns by looking at nutritional factors such as protein, iron, manganese, and calcium. They utilized machine learning models, including Gradient Boosted Trees, Random Forest, K-Nearest Neighbors (KNN), Support Vector Machine (SVM), and Logistic Regression, as well as combined models, to enhance prediction accuracy. Roy and Protity (2023) employed image processing and machine learning to detect irregularities in hair and scalp, including diseases such as alopecia, psoriasis, and folliculitis. For the classification method, a CNN was employed, with the support of pre-processing steps such as histogram equalization and data augmentation to enhance accuracy. Sultanpure et al. (2024) presented a deep learning model using CNN combined with a Django-based web platform to diagnose diseases. Their approach, which employed trichoscopy images, achieved a 91.1% validation accuracy in targeting alopecia. Chowdhury et al. (2024) conducted a deep learning model comparison between VGG19, Xception, Inception, ResNet, and DenseNet for disease classifications on hair and scalp. To assist in the interpretation of the diagnostic tasks in dermatology, Grad-CAM and saliency maps were used (Chowdhury et al., 2024). Khan and Subramaniam proposed a cellular automaton-based rough set neural network with the name of CS-CNS for this task (Khan and Subramaniam, 2023). The authors merged the Cellular Automaton-based Rough Set Theory (CA-RST) Method to improve the performance of the CNN. Khatun et al. used a survey to identify the hair fall patterns in the Bangladeshi community with the help of an ML study (Khatun et al., 2022). They utilized SVM, Naive Bayes, Decision Tree, Random Forest, and XGBoost as the models of comparison. Sajid et al. conducted an extensive survey that unveiled patterns of hair care practices and horological patterns (Sajid et al., 2022). They found out that the female population was more prone to hair fall in comparison with the male population due to their exercise regimen. Naga Sai Chennu et al. performed a comparative analysis of individual algorithms and an ensemble method in their study (Sai et al., 2023). They worked with various models, including SVM, K-NN, decision tree, random forest, and logistic regression. Through their ensemble-based approach, the authors observed that this method outperformed all other individual methods, resulting in higher precision and recall rates. Through this study, the authors have effectively presented the possibility of uniting models to get the correct diagnostic results for the hair fall-related conditions. Moreover, the work mainly relied on organized data and tabular features and had no link to real-time or image-based scalp diagnosis. Therefore, it was not all that relevant in the field of dermatology. In addition, the data used were private, which therefore does not support the generalization needed to obtain the results. Pandikumar et al. (2024) proposed a deep learning framework combining genetic data and scalp health metrics, demonstrating the importance of integrating multimodal inputs for personalized hair loss prediction. Srinivasan et al. (2023) researched stress-induced hair loss with the help of KNN and other ML techniques. Their model also integrated the impact of psychological factors such as depression, anxiety, and attention deficit disorder (Srinivasan et al., 2023). Daniels et al. (2021) carried out a proof-of-concept study that explored the use of artificial intelligence in evaluating hair assembly features only in terms of their capability to be applied to the field of hair care treatments (Daniels et al., 2021). The study compared AI algorithm results with expert human evaluations, revealing that machine learning techniques could be highly beneficial in cosmetic science, as they can provide unbiased and reproducible results, particularly for distinguishing between treated and untreated hair samples. Although this study is an important example of the advantages of applying AI in cosmetic product development, its scope was limited to the product's visual aspects, leaving the analysis somewhat superficial. The study did not delve into examining underlying features or linking them to deeper clinical conditions such as scalp health, follicular integrity, or disease progression. Table 1 details how they applied machine learning, image-based methods, ensemble models, clinical areas, and real-time applicability.

**Table 1:** Comparative analysis of hair loss research studies based on technological and clinical attributes.

**Study/author(s)**	**Uses ML/DL**	**Image-based analysis**	**Ensemble models**	**Clinical focus**	**Real-time applicability**
Farooq et al. (2024)	✓	×	✓	✓	×
Roy and Protity (2023)	✓	✓	×	✓	×
Sultanpure et al. (2024)	✓	✓	×	✓	×
Chowdhury et al. (2024)	✓	✓	✓	✓	×
Khan and Subramaniam (2023)	✓	✓	×	✓	×
Khatun et al. (2022)	✓	×	✓	✓	×
Haag and Chennu (2023)	✓	×	✓	✓	×
Srinivasan et al. (2023)	✓	×	×	✓	×
Hadshiew et al. (2024)	✓	×	×	✓	×
Trueb (2021)	✓	✓	✓	✓	✓
Behal et al. (2024)	✓	✓	×	✓	×
Suryawanshi et al. (2024)	✓	✓	×	✓	×
Chang et al. (2020)	✓	✓	✓	×	✓
Rahman et al. (2024)	✓	×	×	✓	×
Aditya et al. (2022)	✓	✓	×	✓	×
Hafsa et al. (2025)	✓	×	×	✓	✓
Daniels et al. (2021)	✓	✓	×	×	×
Kim et al. (2022)	✓	✓	×	✓	✓
Ho et al. (2023)	✓	×	×	✓	×
Esfandiari et al. (2012)	✓	×	×	✓	×
Pillai et al. (2021)	×	×	×	×	✓
Elsworth and Guttel (2020)	×	×	×	×	✓
Prater et al. (2024)	×	×	✓	×	✓
Lindemann et al. (2021)	×	×	×	×	✓
Lim et al. (2021)	×	×	×	×	✓
Coccomini et al. (2024)	×	✓	×	×	✓

### 2.2 Conventional insights into hair loss without AI integration

Ina M. Hadshiew et al. (2024) commented on the psychological and emotional aspects of hair loss, with a focus on the stress-induced Telogen Effluvium (TE) and Androgenetic Alopecia (AGA) types. The data gathered from the investigation gave information about the influence of chronic stress on hair loss, which, in turn, significantly leads to serious psychological problems, such as low self-esteem, anxiety, and social seclusion. They suggested the incorporation of stress assessment and psychological support as part of clinical diagnosis and treatment. One of the study's critiques is its excessive reliance on observational and qualitative methods, which may lead to inadequacies in the use of objective diagnostic tools or data-driven approaches. The research did not utilize any computational models, machine learning techniques, or quantitative biomarker analysis to predict stress levels or the degree of hair loss. Furthermore, the non-availability of large-scale clinical research studies and real-time monitoring equipment necessitated that the authors limit the application and expansion of their results to a very specific area. To address these provided gaps, potential research can utilize the AI-driven selection of diagnostic tools, the use of wearable stress monitors, and multimodal data integration to create an augmented, predictable, and personalized stress-related hair loss management system. In 2021, Trueb published an exhaustive biological review of pattern hair loss (PHL), concentrating on PHL's genetic, hormonal, prostaglandin-related, and epigenetic causes (Trueb, 2021). The trends that push the condition of pattern hair loss forward in both sexes and the treatments of antiandrogens and their mechanisms were discussed by the contributors. Even though their review is prevailing in the field of PHL, it is a great source of information that is lacking. The study is notably beneficial due to its lack of computational modeling, predictive analytics, or integration with the latest AI-based diagnostic tools. Moreover, the literature or review paper does not utilize data-centric risk assessment algorithms or real-time assessment of treatment efficacy, which places it more on the basic research side and makes it almost unavailable in clinical deployment and personalized healthcare settings. To overcome these limitations, AI can be combined with patient biology to construct predictive, patient-specific models of hair loss risk.

### 2.3 Deep learning and computer vision in scalp health

For front-facing full-head images, Behal et al. (2024) suggested a 2D classifier that is CNN-based for the prediction of stages of hair loss. The authors of this research intended to develop a tool to be used by medical staff to determine whether the patient has this problem with the help of the prompt (Behal et al., 2024). Suryawanshi et al. (2024) introduced an evolved CNN model inspired by the VGG to diagnose dandruff and three types of hair diseases, namely fungal infections and alopecia. The authors have resorted to deep learning, not only for imaging but also for image processing, to increase the accuracy of disease classification (Suryawanshi et al., 2024). Chang et al. (2020) hit on the idea of ScalpEye, a system to check one's scalp conditions through a convoluted neural network-processed camera and a platform that is integrated into the cloud. The invention adopted Faster R-CNN with Inception-ResNet_v2 for disease recognition (Chang et al., 2020). Benhabiles et al. (2019) have completed the first research where deep learning techniques were used for the classification of men's hair loss. The study, as well as the research group, concerned the usage of the Dorban medical classifier in the area of diagnosis (Benhabiles et al., 2019). Rahman et al. (2024) have proposed building an archive of data to be used in the future with CNN and LSTM architectures to be used in a model in the time series for predicting the severity of hair loss for a person who has a family history of hair loss, according to their DNA samples and information about the person's health status. This study examined various machine learning models, including SVM, CNN, and random forest, to evaluate their accuracy rates. Among them, the best-performing model was the CNN model, with 92% accuracy, as reported by (Aditya et al., 2022). Emerging AI Trends and Technological Insights. Hafsa et al. (2025) have highlighted features of the future of AI that will be implemented through the ARTAS robotic system. Moreover, the nanozyme therapy of ML would be a good targeting treatment for the disease. Accordingly, the treatments would take into account comfort and should not be harmful to the patient in any way. The authors have put a special emphasis on the use of these reagent-free therapies (Sheikh et al., 2025).

Daniels et al. (2021) worked on developing AI just to put it into practice for auditing surgical processes, which is partly because the method has been promised to be more efficient than manually raising trails. However, they also used it innovatively; as might be observed, they analyzed hair samples in a new way using the same AI method they had employed before in feature evaluation. To make sure that the AI-based evaluation was really a good method, a comparison of its performance with that of a human expert was conducted (Daniels et al., 2021). The authors, Kim et al., recently conducted a study to locate and estimate the number of hair follicles. A localization step applied YOLOv4 and other detection-transformation models that worked together. Given the results, it was concluded that YOLOv4 was the best choice for this task. More specifically, they concentrated on particular factors such as hair density using a YOLOv4 network (Kim et al., 2022). Ho et al. (2023) studied a disease known as pattern loss. They have established that females predominantly suffer from this disease. They also shed light on how genetic differences in male and female sex could lead to hormone production. Consequently, it is necessary to conduct further studies to confirm the hormonal influence on the disorder. The study also informs us that it is easier to find a solution to certain hair losses with a proper identification of the hormonal processes that can be changed (Ho et al., 2023). Trueb's (2021) research presents a comprehensive discussion of the causes of genetic and hormonal disorders associated with hair loss types, also exploring the use of long-term antiandrogens for scalp coverage (Trueb, 2021). Gezici and Sefer (2024) introduced a deep transformer-based model that predicts asset prices and directions, proving the ability of the model to uncover long-range temporal dependencies and complex multi-feature interactions in financial time-series data. In a similar vein, Tuncer et al. (2022) integrated a deep 2D transformer with convolutional neural networks (CNNs) for the forecasting of asset prices, in which the transformer's attention mechanism represented the temporal relationships and the CNN extracted the spatial patterns from the transformed time-series inputs. Despite being developed in the finance domain, both studies mention the adaptability and the efficacy of the transformer architectures for the analysis of sequential data, thus indicating their significance for our use of transformers in the modeling and forecasting of hairfall-related temporal patterns(Gezici and Sefer, 2024; Tuncer et al., 2022).

## 3 Proposed methodology

**Figure 1:**
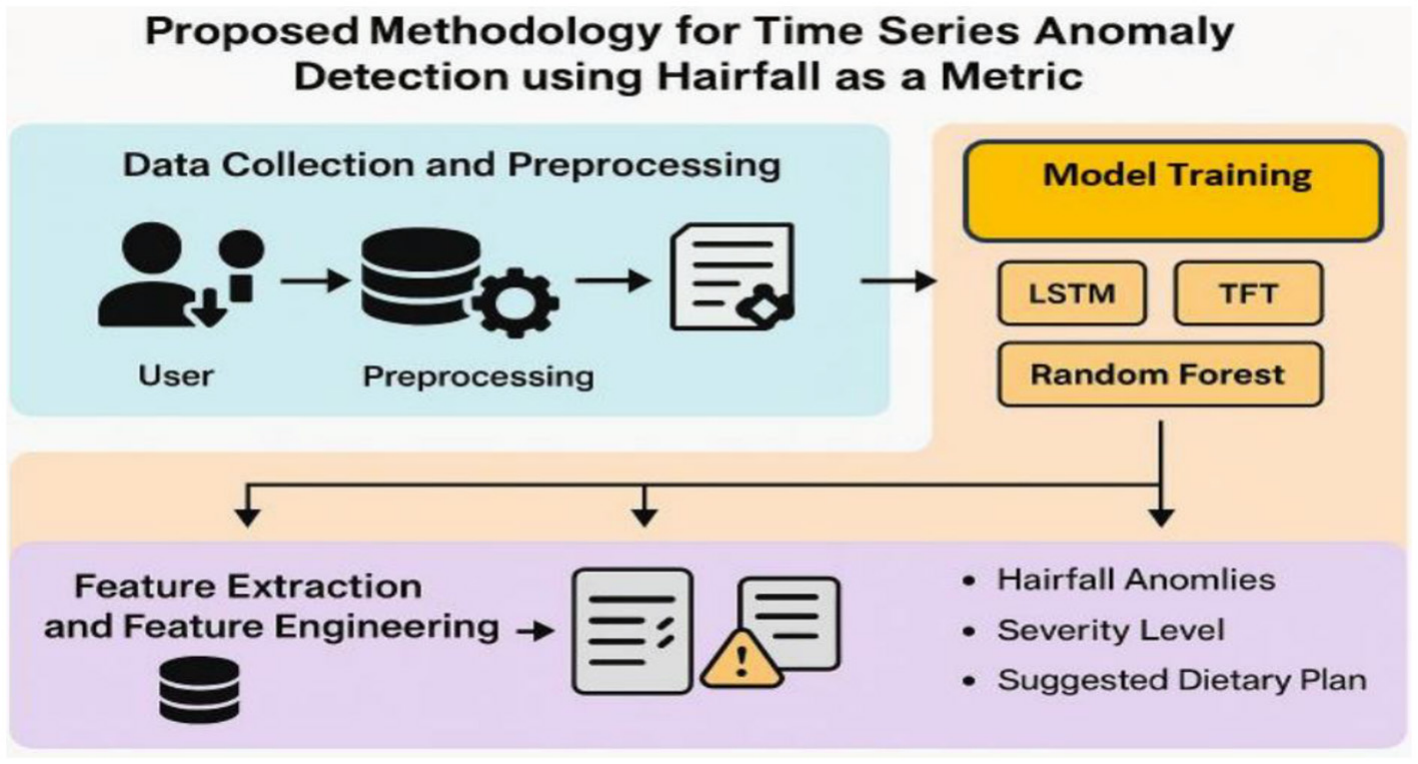
Proposed methodology for time series anomaly detection using hairfall as a metric. The framework comprises user data collection, pre-processing, model training (LSTM, TFT, Random Forest), and feature engineering to detect anomalies, assess severity, and recommend dietary plans.

This section discusses the overall workflow for time series anomaly detection in hairfall analysis. Primarily, the dataset is collected from users, followed by pre-processing to handle missing values, feature extraction, feature engineering, and other data handling techniques. The processed dataset is then implemented using various algorithms such as LSTM, Temporal Fusion Transformer (TFT), and Random Forest, which are compared with the ARIMAX model to identify the best possible model for predicting hairfall. These deep learning methods not only enable anomaly detection but also detect the severity of the condition, thereby recommending suitable dietary plans for the users. This approach facilitates early detection of potential health issues, indicating the intensity of the problem. Figure 1 outlines the data-driven method to detect hairfall anomalies using machine learning models.

### 3.1 Data collection

**Figure 2:**
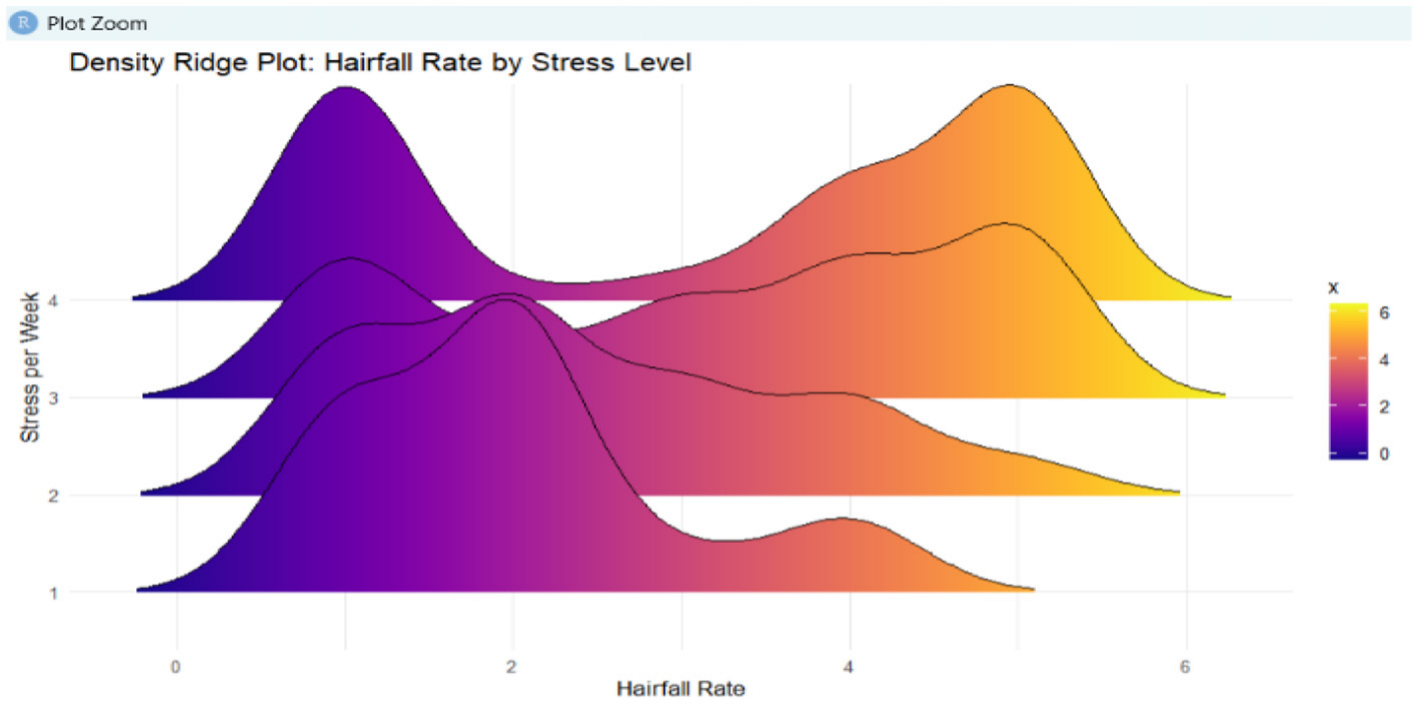
This density ridge plot represents hairfall rate distribution across different stress levels. Gradient coloring indicates frequency density.

Data collection involves collecting the data that supports the prediction of hairfall rate, such as the self-reported hairfall rate, hair strength, dietary habits, lifestyle metrics, and so on, from individuals through surveys and questionnaires. The prediction is solely based on the real-time data collected through a survey. These records are used to analyze and inform decisions by implementing machine learning approaches that contribute to reducing the hairfall rate. Figure 2 illustrates the distribution of hairfall rates categorized by stress levels using a density ridge plot.

#### 3.1.1 Dataset description

Our approach utilizes a real-time dataset where the data collected from nearby individuals captures a comprehensive range of variables related to hair health, lifestyle, diet, stress, and environmental exposure that have a direct impact on hairfall rates. The dataset includes demographic details, such as age and gender, as well as biological factors, including hereditary conditions. Core hair-related features include indicators of hairfall rate, hair thickness, hair density, hair type, scalp condition, and usage of hair treatments and conditioners. Lifestyle attributes include sleeping hours, hair washing frequency, and the type of shampoo used. A significant portion of the dataset focuses on psychological and stress-related metrics, such as daily stress levels and weekly stress exposure.

*HFR*_*i*_= Hairfall rate for individual *i*
*HFI*_*i*_= Hormonal Fluctuation Index (HFI1) for individual *i*
*X*_*i*_ = [*x*_*i*1_, *x*_*i*2_, …, *x*_*in*_]= vector of other features (e.g., nutrient deficiency, stress level, protein intake, etc.)
β = [β_0_, β_1_, …, β_*n*_]= model coefficients
The relationship between hairfall rate and influencing variables can be modeled as:
$HFR_{i}=\beta_{0}+\beta_{1}\cdot HFI_{i}+\overset{n}{\underset{j=2}{\sum }}\beta_{j}\cdot x_{ij}+\;\epsilon_{i}$,
where
β_0_ is the intercept,
β_1_ captures the effect of hormonal fluctuation,
ϵ_*i*_ is the error term capturing unexplained variance.

**Figure 3:**
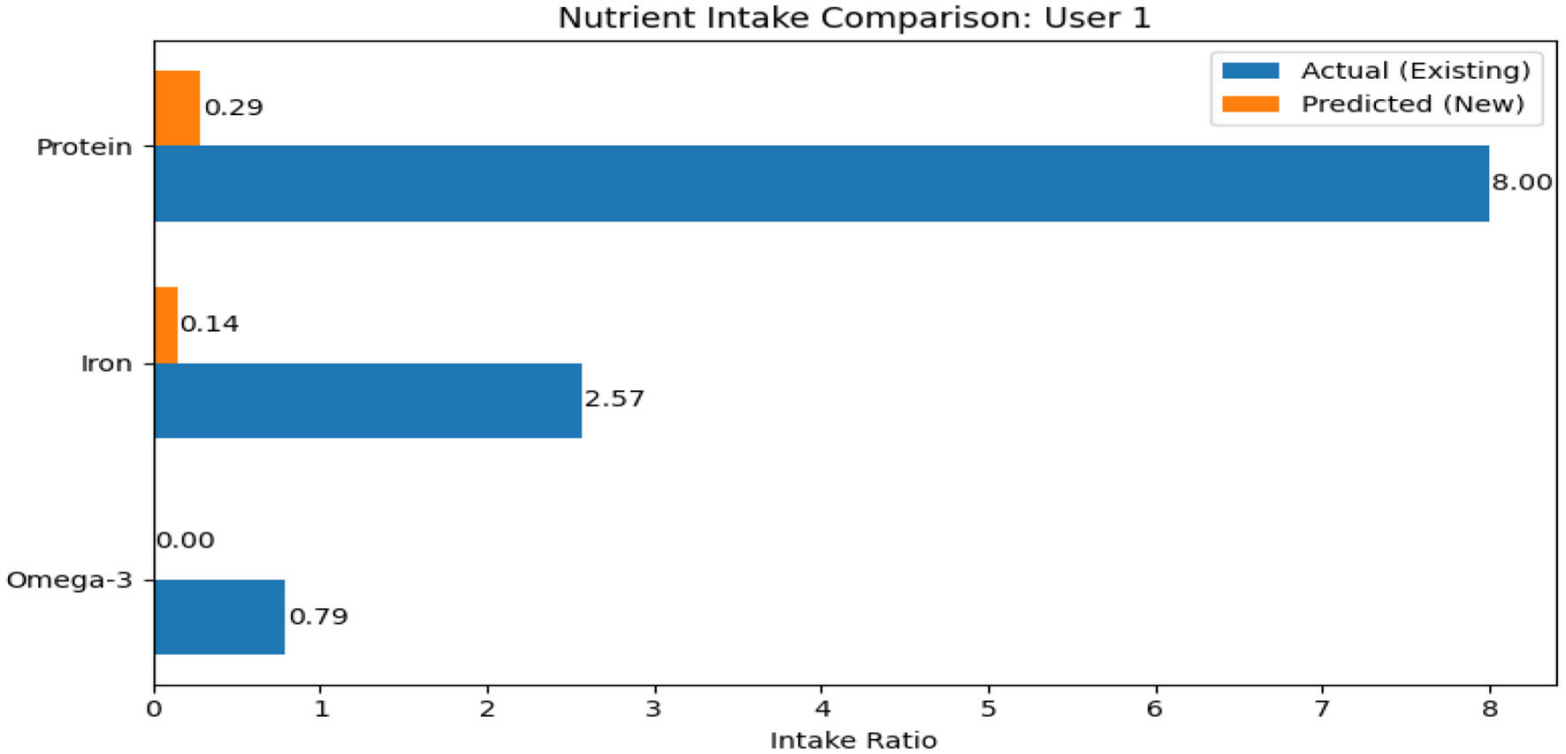
If the user is nutrient deficient, this plot is displayed to indicate the ranges of deficiency by comparing actual intake (blue) with the recommended intake (orange) for Protein, Iron, and Omega-3.

Nutrition-related data are also collected with self-reported intake of protein, iron, and omega-3 fatty acids. Indicators of dietary habits, such as the presence of deficiencies, supplement use, and junk food consumption, are included as well. A comparative analysis of actual and predicted nutrient intake for a sample user is shown in Figure 3. Several derived attributes, including hormonal fluctuation (HFI1), nutrient deficiency score, scalp health score, and stress impact scores, are calculated to support effective prediction. Overall, this balanced dataset provides a rich foundation for analyzing the multifaceted causes of hair loss, integrating biological, environmental, and behavioral dimensions.

- *P*_*i*_= Protein intake (g)
- *I*_*i*_= Iron intake (mg)
- *O*_*i*_= Omega-3 intake (g)
- *J*_*i*_= Junk food consumption indicator (binary or score)
- *S*_*i*_= Supplement use indicator (binary)
- *D*_*i*_= Nutrient deficiency score
- *HFI*_*i*_= Hormonal Fluctuation Index
- *SHS*_*i*_= Scalp Health Score
- *SIS*_*i*_= Stress Impact Score
- ϵ_*i*_= Error term

Then, the hairfall rate prediction function can be written as follows:


$$\begin{array}{c} HFR_{i}=\alpha_{0}+\alpha_{1}P_{i}+\alpha_{2}I_{i}+\alpha_{3}O_{i}+\alpha_{4}J_{i}+\alpha_{5}S_{i}+\alpha_{6}D_{i}\\ +\alpha_{7}HFI_{i}+\alpha_{8}SHS_{i}+\alpha_{9}SIS_{i}+\epsilon_{i} \end{array}$$

Interpretation:

- α_1_, α_2_, α_3_ : Capture the positive or negative influence of essential nutrients
- α_4_ : Models the impact of junk food (likely positive on HFR—more junk, more hairfall)
- α_5_ : Captures the protective effect of supplement use (likely negative on HFR)
- α_6_ : Represents a composite nutrient deficiency score affecting hair health
- α_7_, α_8_, α_9_ : Represent derived health indices related to hormone, scalp, and stress

**Figure 4:**
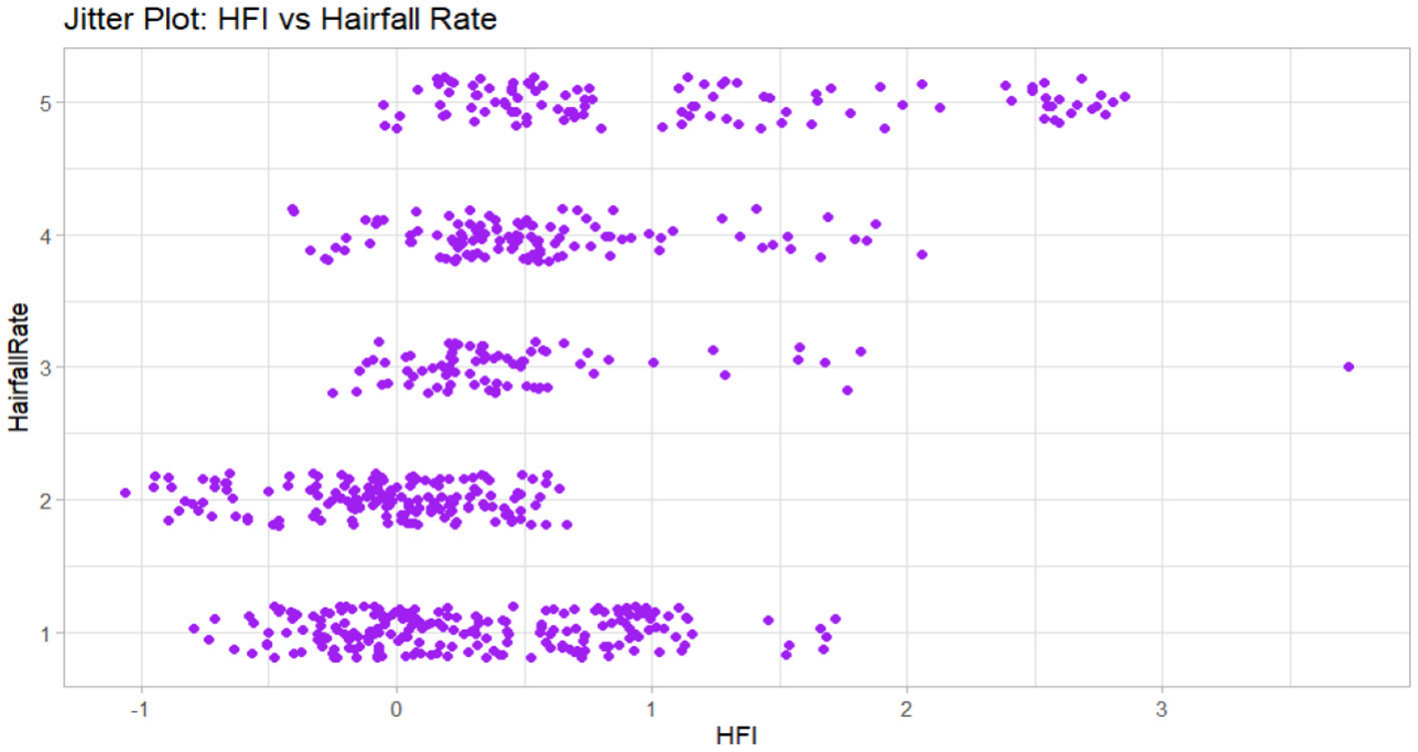
This jitter plot shows the relationship between hairfall rate and HFI (Hormonal Fluctuation Index). Each point represents an individual data entry with vertical jitter to reduce overlap.

Table 2 summarizes the key variables included in the balanced hairfall dataset, categorized into demographic, clinical, behavioral, nutritional, and computed risk dimensions. As illustrated in Figure 4, the jitter plot shows the distribution of Hairfall Rate across different HFI (Hairfall Factor Index) values, highlighting variability and clustering across levels of shedding severity.

**Table 2:** Categorization of variables in the balanced hairfall dataset.

**Category**	**Variable**
Demographic information	Age
	Gender (inferred via gender_protein2, gender_iron2)
Hair fall indicators	Hairfall problem (binary)
	Hairfall rate/day (1–5)
	Hair density (1–10)
	Hair strength (1–10)
	Hair type
	Scalp condition
	Hair thickness (1–5)
	Hair treatment
Lifestyle and behavioral factors	Sleeping hours
	Hair washing frequency
	Shampoo type
	Conditioner usage
	Hereditary hair conditions
Stress and mental health	Experience of hair loss during stress
	Weekly stress frequency
	Stressor type (Exams, Professional, etc.
	Daily stress level (1–10 scale)
Nutritional intake	Protein intake (g/day)
	Iron intake (mg/day)
	Omega-3 intake (g/day)
	Nutrient deficiency
	Water intake
	Vitamin deficiencies (self-reported)
Computed risk scores	Supplement usage
	Created Scalp Score
	Stress Impact Score
	Hair Fall Index (HFI1)

### 3.2 Data pre-processing

#### 3.2.1 Data cleaning

The collected data reflects some unstructured and missing values that contribute to or degrade the model accuracy and prediction rates. To overcome these challenges, data cleaning is necessary and can be achieved by replacing missing values with central tendency measures, such as the mean, median, and mode, which reduces the potential bias created by missing entries. Along with standard deviation, it was used to assess the variability within numerical features and to identify potential outliers, which could also affect model robustness if left unaddressed. To further enhance data consistency and improve algorithm performance, z-score normalization and min-max scaling are used to refine the best out of the data collected.

#### 3.2.2 Data encoding

Encoding techniques, such as ordinal, midpoint, and binary encoding, are used to replace categorical variables with numerical values, enabling effective processing that supports accurate predictions. To categorize hairfall problems, supplement consumption, conditioner used, and diet routines with Boolean values (true and false) into 0s and 1s using binary encoding. Furthermore, midpoint encoding categorizes water intake within a range of values, with the corresponding midpoint calculated. Ordinal encoding approaches categorize several variables that have clear increasing ranks, such as sleeping hours, hair type, shampoo type, stress frequency, and hereditary conditions, into numerical values. This approach withholds the inherent hierarchical relationships within the data, enhancing the model's ability to learn patterns proactively. These encoding strategies contributed significantly to optimizing data quality and model accuracy during the pre-processing phase.

#### 3.2.3 Data featuring

The dataset is further enhanced with additional features that enable individuals lacking domain-specific knowledge to perform sophisticated analysis of hairfall strands. This feature engineering includes the construction of additional features, ensuring relevant attributes are extracted using feature extraction methods to derive meaningful insights from existing data collected. Feature extraction using Pearson coefficients is performed to determine attributes that have the highest correlation with the target variables. These target variables can be hormonal fluctuations, nutrient deficiency, and stress impact score that directly influence the hairfall rate on a severe basis. Their dependencies on existing attributes add relevance to the feature engineering for better analysis.

#### 3.2.4 Data modeling

In this research, multiple linear regression was used to correct scalp health conditions, which have a direct influence on hairfall rates, due to the presence of multiple dependency factors, which are identified using feature extraction, such as shampoo type, conditioner used, and hair_strength This approach, using multiple linear regression, outperforms simple linear regression that fails to capture the complexity of this problem through dynamic weight adjustments. The model considers Pearson correlation coefficients to learn about relationships between features and proportional weights among each contributing attribute. However, when calculating HFI values through feature engineering, this multiple linear regression fails to maintain data integrity, as it involves manual assumptions made through successive iterations to achieve a better fit. Being time-consuming and to avoid assumptions, this model was subsequently eliminated as the ultimate predictor.

- *y*_*i*_ : target variable (e.g., Scalp Health Score or Hairfall Rate)
- *x*_*ij*_ : the *j*^*th*^ feature for sample *i* (e.g., shampoo type, conditioner used, hair strength, etc.)
- β_*j*_ : coefficient for feature *j*
- ϵ_*i*_ : residual error

The model is expressed as follows:


$$\begin{array}{l} y_{i}=\beta_{0}+\overset{n}{\underset{j=1}{\sum }}\beta_{j}x_{ij}+\;\epsilon_{i}, \end{array}$$

where the weights β_*j*_ are optimized by minimizing the mean squared error (MSE):


$$\begin{array}{l} \text{MSE}=\frac{1}{N}\overset{N}{\underset{i=1}{\sum }}{\left(y_{i}-{\hat{y}}_{i}\right)}^{2} \end{array}$$

However, due to:

- Feature interdependence (multicollinearity),
- Need for manual assumptions in feature engineering (e.g., computing HFI),
- Inability to capture non-linearities and complex interactions

MLR fails in this scenario as it compromises data integrity and model interpretability. To achieve a more transparent and optimized prediction, the symbolic regression method was employed, which determines the best-fit mathematical equation for the problem rather than relying on predefined equations or weight tuning. This enables us to generate a usable prediction formula, which is crucial for medical validation and interpretability. Other models, such as decision trees or gradient boosting machines (GBM), were found unsuitable, as they depend on if-else conditional branching that has no standardized formulation for execution, thus proving unrealistic in medical or nutritional prescription contexts. Advanced models, such as XGBoost and neural networks, which act as black-box algorithms, have hidden cases with internal logic and decision paths that are unclear. Therefore, these models are not amenable to deriving meaningful interpretations or validation.

#### 3.2.5 Data balancing

To ensure balanced feature representation, data balancing techniques were employed to identify the inconsistencies in the distribution of key variables. Here, the multiple linear regression technique is employed to model the relationship between the predictor variables and the scalp score, thereby ensuring an accurate estimation of attribute weights. In particular, the focus lies on refining the Scalp Health Score, which exhibits significant variability and is also easily influenced by various factors. The Expectation-Maximization (EM) algorithm was chosen as a refinement mechanism for the situation of missing or uncertain data. The correction is achieved through the iterative estimation of the most probable values by the EM method, which are drawn from the established statistical distributions, thereby leading to the capacity of more conclusive predictions even in the event of partial data input. The EM algorithm makes the probability distribution less erratic as it aims at converting the expected values of scalp condition into a more realistic form, and the integration into the model is much smoother since it does not distort the interpretability of the model. In summary, this solution, which balances the data and estimates the probability, enables the necessary speed for predictions to be made while maintaining the integrity of the individual metrics within the overall hairfall analysis. Let:

- *SHS*_*i*_= Scalp Health Score for individual *i*
- *X*_*i*_ = [*x*_*i*1_, *x*_*i*2_, …, *x*_*in*_]= vector of predictor features (e.g., shampoo type, conditioner use, stress level, and protein intake)
- β = [β_0_, β_1_, …, β_*n*_]= regression coefficients
- ε_*i*_= residual error

Then the scalp health score is estimated as follows:


$$\begin{array}{l} SHS_{i}=\beta_{0}+\beta_{1}x_{i1}+\beta_{2}x_{i2}+\cdots +\beta_{n}x_{in}+\varepsilon_{i}\; \end{array}$$

Let:

- *Z*_*i*_= latent (missing/unobserved) true SHS value
- *Y*_*i*_= observed (possibly incomplete or noisy) SHS value
- θ= model parameters (e.g., means μ, variances σ^2^)

E-Step (Expectation): Estimate the expected value of the latent variable *Z*_*i*_ given current parameters:


$$\begin{array}{l} 𝔼\left[Z_{i}|Y_{i},\theta^{\left(t\right)}\right]={\hat{Z}}_{i} \end{array}$$

This could involve calculating the following equation:


$$\begin{array}{l} {\hat{Z}}_{i}=\mu^{\left(t\right)}+\rho \cdot \left(Y_{i}-\mu^{\left(t\right)}\;\right), \end{array}$$

where ρ is a weighting factor based on observed data confidence or correlation. M-Step (Maximization): Update parameters θ to maximize the expected log-likelihood:


$$\begin{array}{l} \theta^{\left(t+1\right)}=\arg\underset{\theta }{\max}\underset{i}{\sum }𝔼\left[logp\left(Z_{i}|\theta \right)|Y_{i},\theta^{\left(t\right)}\right]\; \end{array}$$

Typically, the model parameters such as:


$$\begin{array}{l} \mu^{\left(t+1\right)}=\frac{1}{N}\overset{N}{\underset{i=1}{\sum }}{\hat{Z}}_{i},\;\sigma^{2\left(t+1\right)}=\frac{1}{N}\overset{N}{\underset{i=1}{\sum }}{\left({\hat{Z}}_{i}-\mu^{\left(t+1\right)}\right)}^{2}\; \end{array}$$

After convergence (i.e., when θ^(*t*+1)^ ≈ θ^(*t*)^), the refined scalp health score is:


$$\begin{array}{l} SHS_{i}^{*}={\hat{Z}}_{i}\; \end{array}$$

This output is now balanced, estimated, and suitable for downstream predictive modeling or anomaly detection.

#### 3.2.6 Data augmentation and SMOTE analysis

The initial dataset had only a small number of samples, which made it more likely to encounter problems such as underfitting (failing to learn effectively), biased results, and poor prediction accuracy. To address this, data augmentation methods were employed to expand the dataset size and introduce variety while preserving the original patterns. Additionally, the Synthetic Minority Over-sampling Technique (SMOTE) was employed to balance the dataset by generating additional instances, thereby balancing the underrepresented classes. Furthermore, SMOTE functions are performed by interpolating between existing data points to create realistic data points that help stabilize the dataset. These pre-processing strategies enhance the model's ability to learn from a more representative distribution, thereby improving predictive accuracy and enhancing reliability in forecasting hairfall rates.

## 4 Architecture

This architectural diagram depicts the overall process of anomaly detection using machine learning techniques. Primarily, the data acquisition phase involves identifying the problem to understand the scope of analysis and the challenges it presents. This is followed by a detailed literature survey to gain a better understanding and gather existing insights from studies, white papers, journals, and publications, analyzing the methods and data sources used. This facilitates the efficient collection of real-time data through well-structured and relevant questionnaires. The next phase is the data pre-processing layer, which handles missing values using appropriate central tendencies and normalization techniques to maintain data uniformity. This leads to the extraction of meaningful characteristics to model features by using suitable feature extraction techniques, forming structured data for analysis. This makes the data suitable for the next phases of machine learning model application and evaluation.

The model application layer phase, followed by pre-processing, involves the identification of machine learning algorithms for implementation. Models that deal with time-related data, such as LSTM, TFT, and ARIMAX, as well as those that engineer features from time series, combining static and dynamic factors, such as Random Forest, are selected. These are capable of modeling sequential patterns, handling dynamic variables, and capturing fluctuations over time. These models are evaluated over metrics such as R-squared score, mean squared error, accuracy, and precision. Once the models are validated and selected hierarchically, the model construction phase involves building a new algorithm using the selected model from the previous phase, making valid predictions. To assess the algorithm, the Severity Assessment Layer phase checks the seriousness or intensity of the detected anomalies. It is performed by threshold fixation, which distinguishes between normal and concerning conditions, raising alerts if the severity is experienced.

This phase identifies and evaluates candidate models for time-series-based hairfall prediction. Let:

- **X**_*t*_ = [*x*_*t*1_, *x*_*t*2_, …, *x*_*tn*_] : observed time-series input at time *t*
- Z: static features (e.g., gender, age, genetics)
- M : a candidate model (e.g., LSTM, TFT, ARIMAX, and Random Forest)

The general model function becomes:


$$\begin{array}{l} {\hat{y}}_{t}=M\left({\text{X}}_{t-p:t},\text{Z}\right),\;where\;p\;is\;the\;time\;window\;size \end{array}$$

Model performance is evaluated using:

- *R*^2^ Score:

$$\begin{array}{l} R^{2}=1-\frac{\sum_{t}{\left(y_{t}-{\hat{y}}_{t}\right)}^{2}}{\sum_{t}{\left(y_{t}-ȳ\right)}^{2}} \end{array}$$

- Mean Squared Error (MSE):

$$\begin{array}{l} \text{MSE}=\frac{1}{T}\overset{T}{\underset{t=1}{\sum }}{\left(y_{t}-{\hat{y}}_{t}\right)}^{2} \end{array}$$

- Accuracy/Precision/Recall for classification-based severity outputs.

Lastly, the Recommendation and Dashboard Layer simply makes what has been done clear and understandable by the model. The layer attempts to identify common patterns while also tracking the occurrence of anomalies across the data and suggesting diet or health-related tips. In other words, this sequence of parts constitutes a smooth transition from the stage of data collection to the conclusion of personalized recommendations.

Once the best model $M^{*}$ is selected, it is refined and used for predictions.


$$\begin{array}{l} {\hat{y}}_{t}=M^{*}\left({\text{X}}_{t-p:t},\text{Z}\right) \end{array}$$

The Severity Assessment Layer defines whether the predicted value ${\hat{y}}_{t}$ is normal or critical. Let:

- τ : Severity threshold
- *S*_*t*_ : Severity status

$$\begin{array}{l} S_{t}=\{\begin{array}{lll} 1 & if\;{\hat{y}}_{t}\geq \tau & \text{(critical)}\\ 0 & \text{otherwise} & \text{(normal)} \end{array} \end{array}$$

An alert is triggered when *S*_*t*_ = 1. In the Recommendation and Dashboard Layer

If *S*_*t*_ = 1, the system triggers a personalized response function *R*(·) based on user profile **Z** and history **X**_1:*t*_ :

Recommendation_*t*_ = *R*(**Z**, **X**_1:*t*_)

Examples of *R*(·) could be:

- Recommend iron-rich foods if iron intake < θ_1_
- Suggest scalp therapy if the Scalp Health Score < θ_2_
- Recommend meditation if the Stress Impact Score > θ_3_

**Figure 5:**
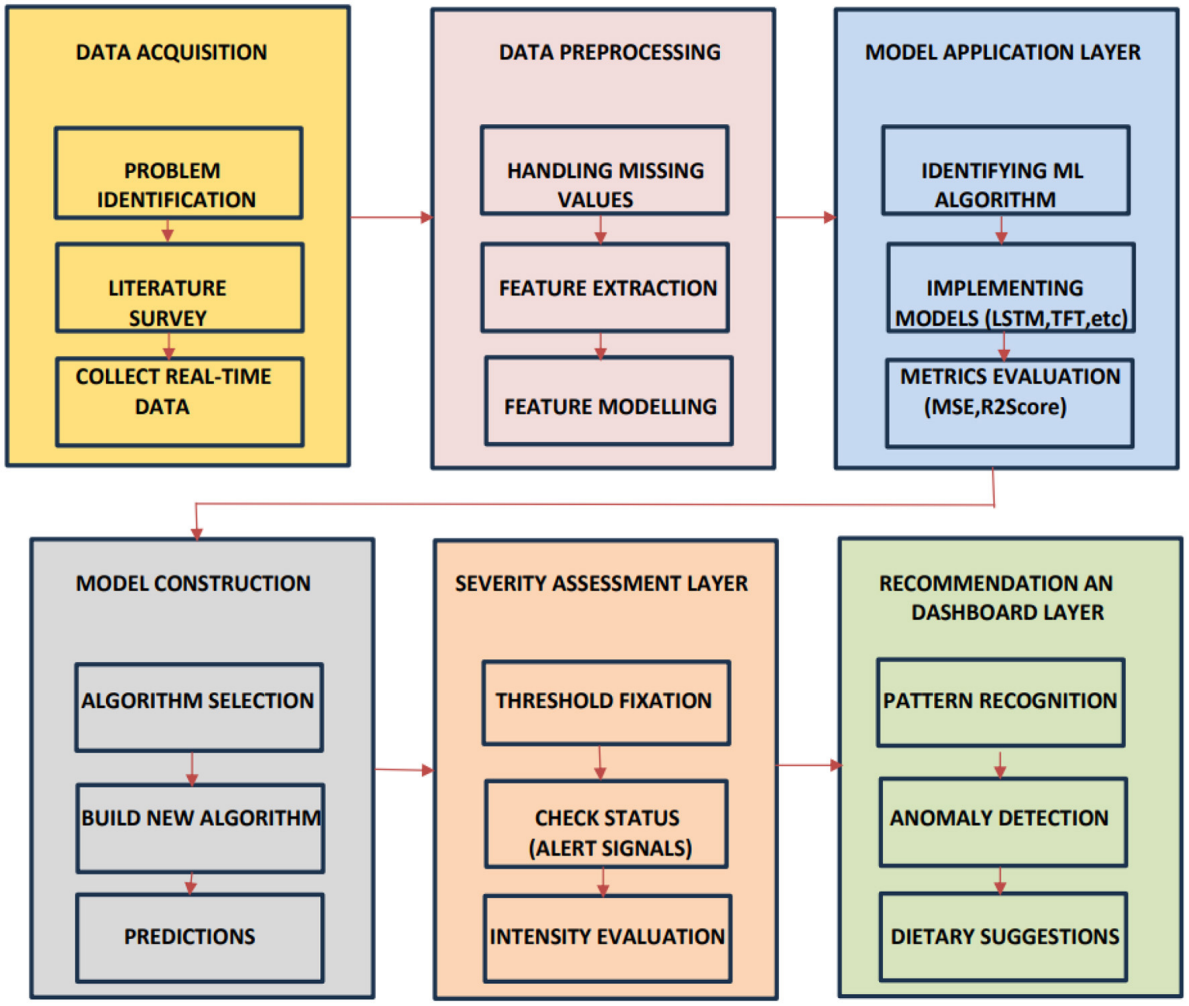
illustrates a comprehensive predictive analytics framework encompassing data acquisition, pre-processing, model development, severity assessment, and recommendation generation. The integrated workflow enables accurate predictions, performance evaluation, and the delivery of actionable insights through a structured, multi-layered process.

Figure 5 displays the complete configuration of the hair fall detection and recommendation framework and its working mechanism. The diagram demonstrates a modular pipeline that encompasses the aforementioned stages of working, including data collection, pre-processing, model building, severity categorization, and dietary requirement identification through anomaly detection.

### 4.1 Mathematical formulation of Temporal Fusion Transformer (TFT)

The Temporal Fusion Transformer (TFT) is an explainable multi-horizon forecasting model that integrates recurrent layers, attention mechanisms, and gating modules to manage both static and time-varying inputs.

Let:

- $x_{t}^{\left(d\right)}$ : dynamic covariate at time *t*
- *z*^(*s*)^ : static covariate
- *y*_*t*_ : target variable (e.g., Hairfall Rate or HFI)
- ${\hat{y}}_{t+\tau }$ : predicted target at forecast horizon τ

#### 4.1.1 Variable selection via Gated Residual Network (GRN)

For each covariate:


$$\begin{array}{l} v=\text{GRN}(x)=\text{LayerNorm }(x+\text{GLU }(W_{2}\;\cdot \text{ ELU}\left(W_{1}x+b_{1}\right)\\ \;+b_{2})) \end{array}$$

where GLU is the Gated Linear Unit:


$$\begin{array}{l} \text{GLU}\left(a,b\right)=a⊗\sigma \left(b\right) \end{array}$$

#### 4.1.2 LSTM encoder-decoder


$$\begin{array}{l} \begin{array}{c} h_{t}={\text{LSTM}}_{\text{enc }}\left(v_{t},h_{t-1}\right)\\ {\tilde{h}}_{t+\tau }={\text{LSTM}}_{\text{dec }}\left(v_{t+\tau },h_{t}\right) \end{array}\; \end{array}$$

#### 4.1.3 Temporal attention


$$\begin{array}{l} \begin{array}{c} \alpha_{t,i}=\frac{\text{exp}\left(Q_{t}K_{i}^{⊤}/\sqrt{d_{k}}\right)}{\sum_{j}\text{exp}\left(Q_{t}K_{j}^{⊤}/\sqrt{d_{k}}\right)}\\ c_{t}=\sum_{i}\alpha_{t,i}V_{i} \end{array} \end{array}$$

#### 4.1.4 Output layer


$$\begin{array}{l} {\hat{y}}_{t+\tau }=W_{o}\left[{\tilde{h}}_{t+\tau };c_{t+\tau }\right]+b_{o} \end{array}$$

#### 4.1.5 Loss function


$$\begin{array}{l} L=\frac{1}{N}\overset{N}{\underset{n=1}{\sum }}\frac{1}{\tau_{\max}}\overset{\tau_{\max}}{\underset{\tau =1}{\sum }}{\left(y_{t+\tau }^{\left(n\right)}-{\hat{y}}_{t+\tau }^{\left(n\right)}\right)}^{2} \end{array}$$

This architecture allows the TFT to:

- Select the most relevant variables dynamically
- Use recurrent layers for local temporal patterns
- Apply attention to long-term dependencies
- Maintain interpretability through variable and time-step importance scores

Though the above description accurately presents the mathematical operations of the Temporal Fusion Transformer (TFT), it can also be depicted as a simpler explanation. The TFT is a device that resembles a clever helper, taking into account past data to make informed guesses about the future. In the beginning, it decides which input features are the most significant at a certain time. Such features could be, for instance, stress level, protein intake, or hairfall rate. After that, it implements a memory function to remember recent patterns and trends so that relevant historical information will not be lost. Moreover, it has a focus mechanism that draws more attention to particular past periods or occurrences that have the most significant impact on future results. Consequently, it goes through and combines the data, and thus, TFT makes its last prediction of future hairfall rates or the Hormonal Fluctuation Index (HFI). At the end of the day, it measures the error by comparing the predicted results with the actual ones and adjusts the internal parameters to improve accuracy over time. Basically, the TFT picks the most necessary parts, keeps track of the past that was useful, concentrates on the important times, and gains from failures, thus being very efficient in forecasting complex hairfall trend scenarios where lifestyle, nutrition, and hormones play an interdependent role over time.

## 5 Dataset description and derived formulae

This research is based on a dataset that contains information about 750 participants, including university student volunteers and individuals from the nearby community, collected in February and March 2025. The data were collected using structured, self-administered questionnaires designed to record the rate of hair loss, hair quality, lifestyle patterns, dietary habits, stress levels, and sleep schedules. These variables have been used as the main inputs for the models as well as for the calculation of some new features. Since the dataset is highly sensitive and contains personally identifiable information, it is not publicly accessible. However, we provide a thorough account of the variables, the data collection process, and the steps taken in pre-processing, which facilitates the replication of the research with similar datasets.

### 5.1 Stress impact score

To formulate the impact of stress on hairfall rate, a derived attribute, the stress impact score, is calculated using:


$$\begin{array}{r} \text{STRESS IMPACT SCORE }=\text{DAILY STRESS LEVEL/}\\ 7\;\times \text{(STRESS FREQUENCY)} \end{array}$$

This formulation integrates both the intensity (daily stress level) and recurrence (frequency of stress) experienced within a week, where the independent variables are collected from individuals as self-reports. Dividing by 7 normalizes the score to a daily scale, facilitating consistent interpretation across the data. This derived metric measures how effectively the stress impact score predicts hairfall severity. The selection of relevant attributes is derived by performing a feature engineering technique, as it enhances the representation of latent stress-related factors.

### 5.2 Nutrient deficiency score

The nutrient deficiency score is calculated to quantify the impact of key dietary components on hair health. The score is calculated by using the formula:


$$\begin{array}{l} \text{NUTRIENT DEFICIENCY SCORE}\\ =\text{1–(PROTEIN INTAKE + OMEGA-3 INTAKE}\\ +\text{IRON INTAKE),} \end{array}$$

where each nutrient intake was individually computed by the following:


$$\begin{array}{r} \text{NUTRIENT INTAKE}=\text{FREQUENCY OF INTAKE IN A WEEK}\\ {}*\text{BIO-PROFILE SCALER (Per day)}/\\ \text{BIO-PROFILE SCALER (per week)}. \end{array}$$

The Bio-Profile Scaler is the gender- and age-based criteria for understanding dietary supplements. This score estimates the extent of nutritional deficiency based on the intake frequency of three key nutrients essential for hair growth: protein, omega-3 fatty acids, and iron. The formulation of nutrient intake considers overall nutrient intake by a person over a week by calculating the frequency of intake values with respect to the bio-profile scaler divided by the overall nutrient intake an average person should consume in a week (in RDA) as per NIH (National Institute of Health org.), ensuring consistent comparison across the data. These derived metrics serve as an effective composite feature that captures the deficiency of multiple essential nutrients. For instance, protein is critical for maintaining hair structure, and if the intake is not sufficient, it leads to brittle and breakable strands. Iron plays a pivotal role in transporting oxygen to hair follicles, and its deficiency can inhibit follicular strength and growth. Omega-3 fatty acids contribute to scalp hydration and overall follicle health. By combining these into a unified deficiency score, the model gains an enhanced representation of how diet-related risk factors influence hairfall severity.

### 5.3 Scalp health score

The scalp health score, a composite metric, was formulated to assess the condition of the scalp, which significantly influences the hairfall outcomes. The score is derived using a linear regression equation as mentioned below:


$$\begin{array}{r} \text{SCALP\_HEALTH\_SCORE}=\text{W0}+\text{W1}\times \text{SHAMPOO\_TYPE}\\ +\text{W2}\times \text{CONDITIONER USED}\\ +\text{W}3\times \text{HAIR STRENGTH}, \end{array}$$

where w0 represents the bias term and w1, w2, and w3 represent the weights calculated for each factor that contributes significantly to the health of the scalp. All the contributing factors are extracted by feature engineering, where the shampoo type captures the chemical or organic nature of products used, the conditioner used reflects the presence or absence of conditioning treatments, and hair strength accounts for the resilience and structural integrity of the hair strand. By applying linear regression to calculate the scalp health score, which provides a quantifiable approach to scalp and hair care practices, it enables deeper insights into how external treatments affect the hairfall rate. Further, the EM algorithm was performed to calculate the corrected scalp health score for better performance.

### 5.4 Hormonal fluctuation

Hormonal fluctuations are a critical factor influencing the regulation of the hair growth cycle. Any disruption in hormonal balance can lead to excessive hair shedding or thinning. To quantify the Hormonal Fluctuation Index (HFI), the following formula is used:


$$\begin{array}{r} \text{HFI}=\text{(STRESS IMPACT SCORE + DAILY STRESS LEVELS}\\ +\text{HAIRFALL RATE PER DAY – SLEEPING HOURS)}/\\ \text{(PROTEIN RATIO + IRON RATIO + OMEGA RATIO}\\ +\;1), \end{array}$$

where the Stress Impact Score and Daily Stress Level capture psychological stress, the most impactful trigger for hormonal imbalance, the Hairfall Rate per Day contributes as a key factor that best indicates the fluctuation rate, and sleeping hours are subtracted from the index, as insufficient sleep is associated with hormonal irregularities. Additionally, the protein ratio, iron ratio, and omega ratio, which are negatively correlated, are calculated and divided, thereby accounting for dietary contributions to hormonal regulation. Here, +1 is the stabilizing term or smoothing constant that tackles the worst case of nutrient intake, where all three intakes are zero, causing division error. This comprehensive formulation enables the HFI to encapsulate both internal and external influences, making it a valuable feature in predictive modeling.

## 6 Schematic diagram

**Figure 6:**
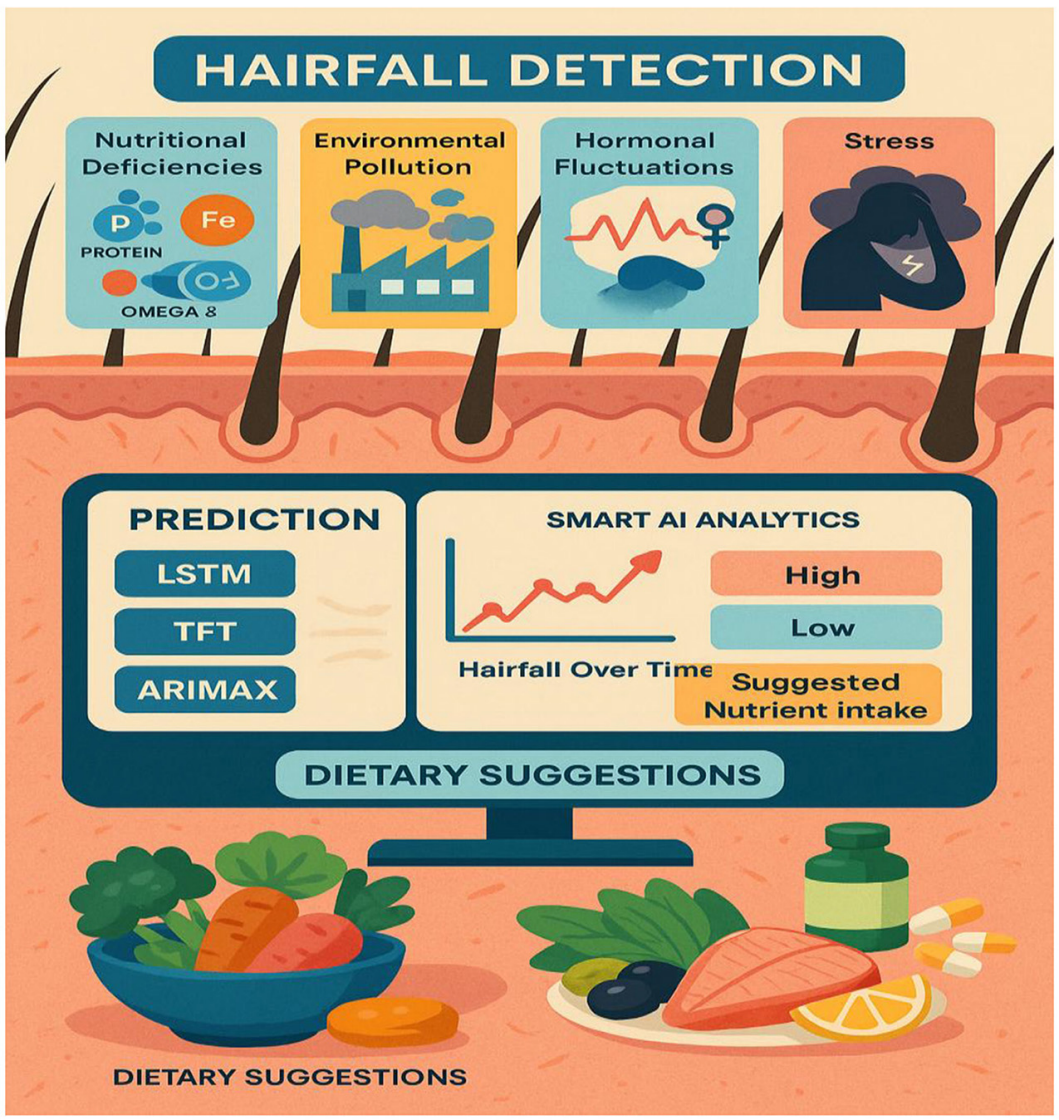
ML-based hairfall detection system that highlights key factors, prediction models, and dietary suggestions.

The schematic diagram depicted in Figure 6 illustrates a machine learning-based hair fall detection system that considers multiple factors contributing to hair loss, including nutritional deficiencies (such as protein, iron, and omega-3 fatty acids), environmental pollution, hormonal fluctuations, and stress levels, to perform analysis. These elements are visually represented as affecting the hair follicles, indicating their direct impact on hair health. The key process involves engineering new ML-powered detective models, which include implementing various models such as LSTM, TFT, random forest, and ARIMAX, specialized in handling time-series data. These models handle trends in hairfall with time and forecast future conditions based on temporal and health-related features. The system further comprises a Smart AI Analytics module that utilizes machine learning techniques to classify the severity of hairfall as either “high” or “low” levels, dynamically mapping over time. The predictive output provides an indication of potential threats, along with suggested dietary plans. Moreover, to some extent, this scientific breakthrough has led to innovative and rapid health improvements resulting from hair conditioning. The basic principle of this prediction is that data science and nutrition science are combined in the direction of hairfall management, which is effective.

### 6.1 Hyperparameter selection for TFT

We conducted a hyperparameter selection process for the Temporal Fusion Transformer (TFT) using a systematic tuning procedure to tailor the model to our hairfall forecasting dataset. First, we utilized the default settings from the original TFT implementation (Lim et al., 2021) and the PyTorch Forecasting library. We then refined our parameters through a grid search combined with validation set performance. Among the hyperparameters that we decided to tune, we could mention hidden layer size, number of LSTM layers, dropout rate, learning rate, attention heads, batch size, and maximum prediction horizon. For each parameter, we tried different values. For instance, it was tested that if the hidden layer size is 64, 128, or 256, 128 would be the best with respect to the balance of accuracy and computation time. We tested learning rates ranging from 1e-4 to 1e-3, and we found that the rate of 3e-4 yielded stable convergence. The tuning operation was 5-fold cross-validation guided, using validation MSE as the main selection criterion and F1 score as an auxiliary measure of classification accuracy. The chosen setup was not only good in training but also on unseen test data, which means that the model was properly optimized for this application.

## 7 Performance analysis

To observe how correctly each of the selection algorithms predicted the extent of the hairfall, based on time-centric user data, a few machine learning and deep learning models, such as LSTM, Temporal Fusion Transformer (TFT), Random Forest, and ARIMAX, were operated. Besides LSTM, Random Forest, and ARIMAX, we also considered two further baselines to verify the robustness of the comparison between our TFT model and the competitive ones. These were a Gated Recurrent Unit (GRU) network. This recurrent architecture can efficiently model complex temporal dependencies, and a Temporal Convolutional Network (TCN), which, through dilated causal convolutions, can capture multi-scale temporal patterns. The reason for choosing these models was that they are particularly suitable for non-linear and multi-feature time-series forecasting, thus offering a more challenging benchmarking of the performance.

The instruments were assessed in terms of several measurements, which include mean squared error (MSE), *R*^2^ score, accuracy, precision, recall, F1 score, and specificity. At this stage, the model's accuracy score reflects how good the model is overall, and the *R*^2^ score shows how well the model can explain the variations of hairfall that can be the input features, like stress, diet, and sleep, which are the main things responsible for verifying the model's predictive ability. Mean Squared Error (MSE) is a metric that squares the average deviation between the actual and the forecasted values, which demonstrates precision in the results. Precision is about correctness and reducing the chances of false alarms in severe cases by indicating how many of the positively predicted classes are indeed correct. The F1 score is a combination of precision and recall and gives a consistent performance indicator. Furthermore, specificity demonstrates how effectively the model identifies non-critical cases, thereby avoiding excessive unnecessary alerts. When taken together, these measurement scales enable us to confirm that the model is accurate. In real time, it will take an important step as the time of the anomaly occurrence arrives, and thus, it will be dependable for early monitoring of hairfall cases. The parameters are illustrated in Equations (1)–(7)


(1) $$\begin{array}{l} \text{Specificity}=\text{TN/(TN + FP)} \end{array}$$

It indicates the fraction of true normal instances that the model is able to recognize accurately. Subsequently, higher specificity implies a lower false alarm rate.


(2) $$\begin{array}{l} \text{Accuracy}=\text{(TP + TN)}/\text{(TP + TN + FP + FN)} \end{array}$$

Accuracy is the quantitative representation of the number of correct predictions out of the total predictions made. It shows the overall correctness of the model.


(3) $$\begin{array}{l} \text{Precision}=\text{TP/(TP + FP)} \end{array}$$

Precision measures the fraction of predicted positive instances that are truly positive. It indicates the number of times that the model's “abnormal” forecasts are accurate in our case.


(4) $$\begin{array}{l} \text{Recall}=\text{TP}/\text{(TP + FN)} \end{array}$$

Recall is a representation of a model's capability to properly find abnormal hairfall instances that are abnormal cases in total.


(5) $$\begin{array}{l} \text{F1 Score}=2\times \left(\text{Precision}\times \text{Recall}\right)/\text{(Precision + Recall)} \end{array}$$

The F1 score blends the precision and recall aspects into one number. It keeps its relevance when working with an uneven class distribution.


(6) $$\begin{array}{l} \text{Mean Squared Error (MSE)}=\text{(1/n)}\times \Sigma {\left(y_{i}--ȳ\right)}^{2} \end{array}$$

MSE quantifies the mean of the squared deviations between the estimated and the actual values of a variable, where these differences indicate the closeness of the predictions to the true values.


(7) $$\begin{array}{l} {\text{R}}^{2}\text{Score}=1-\left[\Sigma {\left({\text{y}}_{i}-ȳ\right)}^{2}/\Sigma {\left({\text{y}}_{i}-ȳ\right)}^{2}\right] \end{array}$$

R^2^ indicates what part of the variance of the dependent variable has been accounted for by the model.

where *TP* and *T N* indicate true positives and negatives, and *FP* and *F N* signify false positives and negatives of the hairfall rate. Y represents the hairfall rate of each sample. The efficiency of the proposed models was analyzed and inferred in Table 3.

**Table 3:** Performance comparison of machine learning models for hairfall prediction.

**Parameters**	**LSTM**	**TFT**	**Random forest**	**ARIMAX**	**GRU**	**TCN**
Accuracy (%)	93.21	97.52	95.54	95.54	94.87	96.12
Precision (%)	93.06	97.44	95.43	58.41	94.75	96.05
Recall (%)	93.21	97.25	95.23	51.15	94.68	95.92
F1-score (%)	93.12	97.29	95.30	52.51	94.71	95.98
Specificity (%)	98.41	99.39	98.89	88.54	98.21	98.95
MSE	0.09	0.03	0.04	0.75	0.06	0.05
*R*^2^ score	0.96	0.98	0.98	0.51	0.97	0.97

Table 3 presents a comparative analysis of various forecasting models based on performance metrics. The results highlight notable variations in accuracy, precision, and generalization capability across the models.

**Figure 7:**
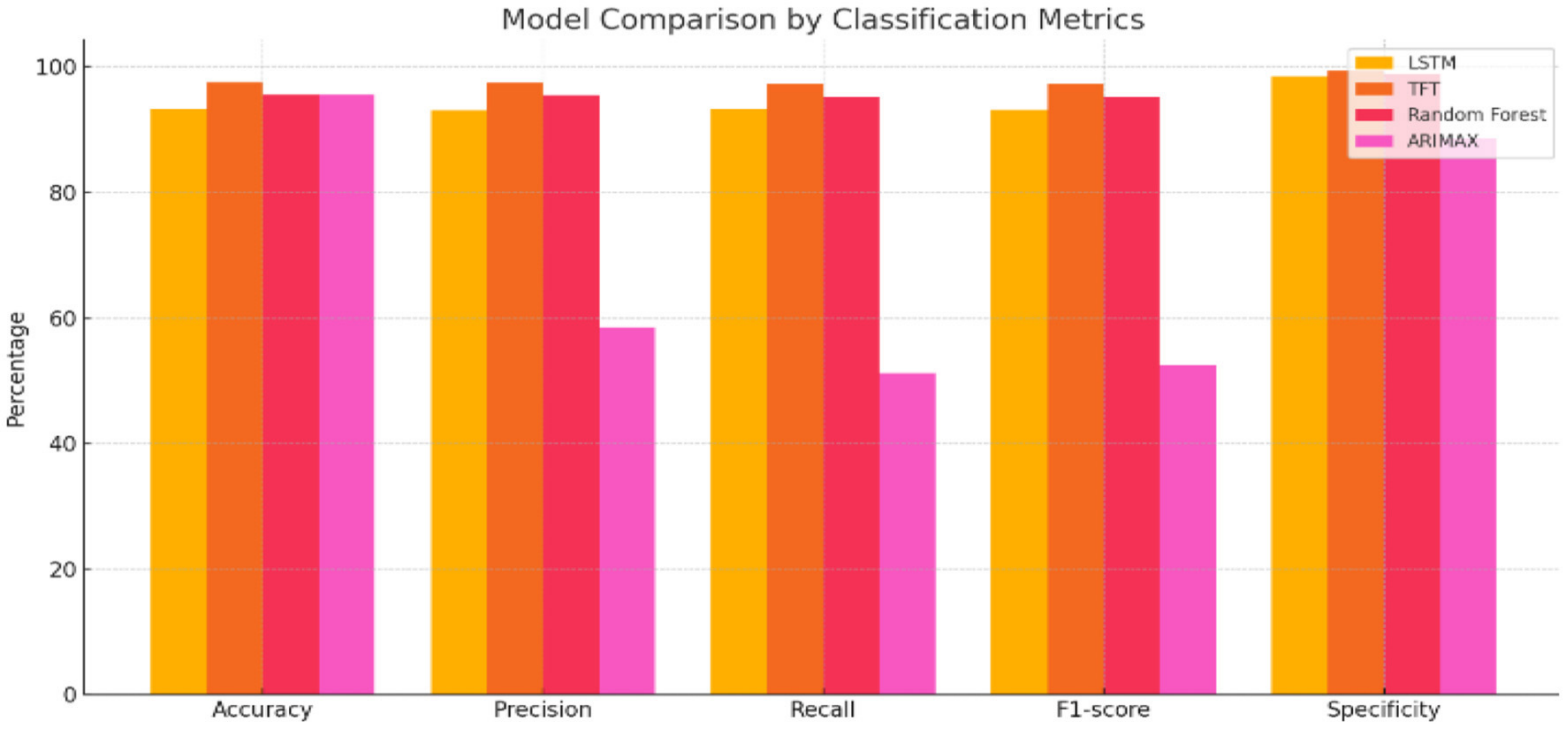
Illustrates the comparative performance of different forecasting models across key classification metrics. The chart highlights significant differences in accuracy, precision, recall, F1-score, and specificity, indicating varied effectiveness and reliability among the evaluated models.

Based on the analysis, we infer that TFT consistently outperformed the other models, achieving the highest accuracy (97.52%), precision (97.44%), recall (97.25%), and F1-score (97.29%), along with the lowest mean squared error (0.03) and the highest R^2^ score (0.98). These results clearly demonstrate the model's robustness across multiple evaluation metrics, confirming its ability to generalize well beyond the training data. These clearly reflect its superlative ability to detect anomalies with minimal false alerts while maintaining solid predictive strength. However, LSTM and Random Forest algorithms followed closely, with LSTM scoring 93.21% in accuracy and Random Forest at 95.54%. Both models showed reliable precision and recall values above 93%, with minimal error margins (MSE of 0.09 and 0.04, respectively). ARIMAX, however, underperformed in comparison—despite matching Random Forest's accuracy (95.54%), its precision (58.41%), recall (51.15%), and F1-score (52.51%) were significantly lower, indicating a poor classification rate and prediction score. The inclusion of GRU and TCN in the baseline comparison further strengthened the evaluation, as these architectures are well-suited for capturing complex temporal dependencies and multi-scale patterns in time-series data. As indicated by Figure 7, the comparison of the accuracy of the four models—LSTM, TFT, Random Forest, and ARIMAX—is presented, and the graphic also shows that the TFT model outperformed the other models in most of the matrices, such as accuracy, precision, recall, F1-score, and specificity.

Therefore, the Temporal Fusion Transformer emerges as the most dependable and precise model for real-time hairfall monitoring and proactive anomaly detection, offering the potential for early intervention through lifestyle and dietary guidance. The results of the GRU and TCN baselines are reported alongside TFT, LSTM, Random Forest, and ARIMAX. To determine whether the observed performance differences were statistically significant, we conducted two-tailed paired t-tests on the evaluation metrics across all models. The results confirmed that TFT's improvements over the next-best-performing baseline were statistically significant (p < 0.05) for both forecasting accuracy and anomaly detection metrics.

## 8 Implementation

Our proposed model focuses on time-series anomaly detection for hairfall by utilizing time-centric data collected from the user to monitor irregularities over time. These sudden spikes or drops in hairfall rate or hormonal fluctuations may indicate underlying health threats. A newly designed algorithm was constructed to accomplish these tasks by integrating the most effective aspects of various pre-existing models. Over performance analysis, the Temporal Fusion Transformer (TFT) demonstrated the highest accuracy and reliability in capturing temporal patterns, making it the primary backbone of the newly constructed hybrid algorithm.

### 8.1 Proposed algorithm


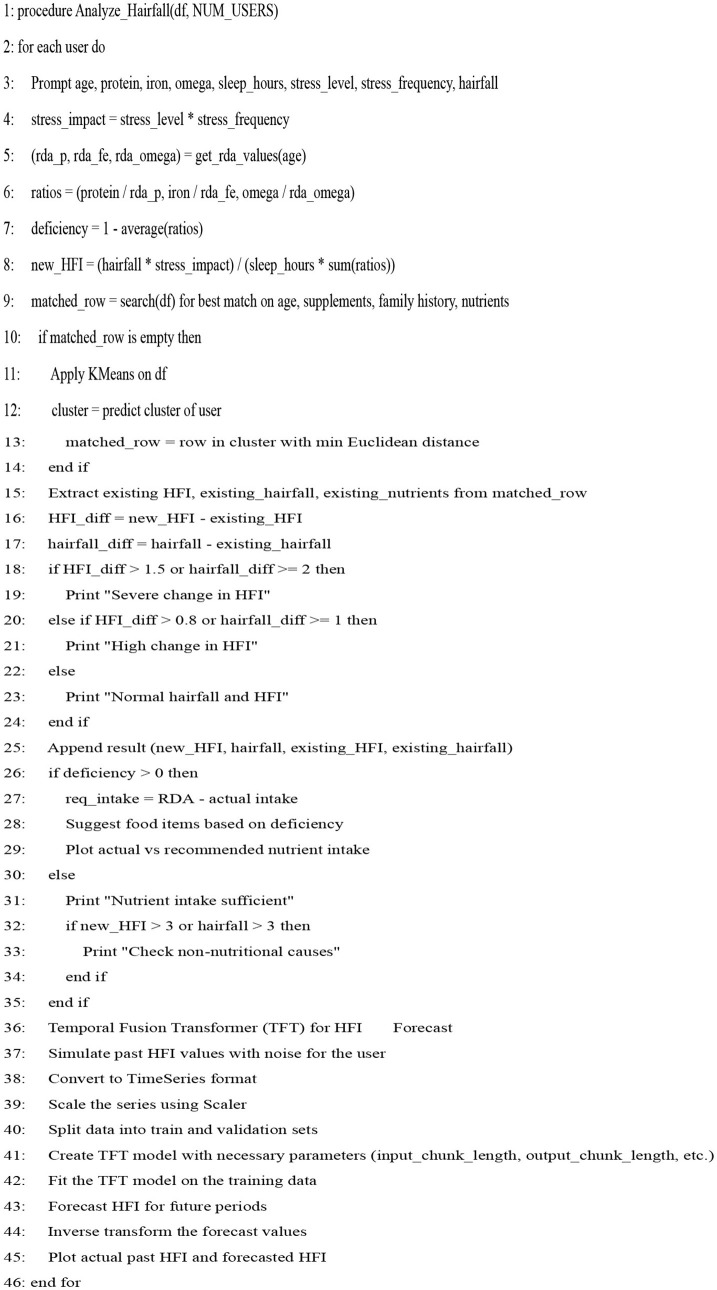


Our model is designed to detect the anomalies that indicate a threat by improving the underlying model (TFT). The algorithm is programmed to take dynamic user inputs and make intelligent comparisons with historical datasets to detect abnormal hairfall patterns. Initially, the user is asked to provide personal information, including age, prevailing hairfall rate (on a scale of 1–5), mean hours of sleep, level of stress and frequency, protein, iron, and omega-3 intake over the past week, supplement usage, and family history of hairfall. The collected input is cross-referenced with a pre-existing dataset to identify matching attributes such as age, nutrient intake, supplement usage, and hereditary factors. If there exists a matching row, the data is directly used for computations; else, the algorithm applies K-means clustering to classify different sets of data as clusters and identify the closest tuple using Euclidean distance.

After the matching phase, the algorithm computes the stress impact score and nutrient intakes, which indicate nutrient deficiency. It updates the user's recent Hairfall Index using appropriate formulas discussed in the section Data Featuring. An anomaly is highlighted if the variation in HFI is more than 1.5 or the hairfall rate exceeds by more than 2 against the matched information. A slight deviation (difference in HFI > 0.8 or difference in hairfall ≥ 1) generates a mild warning if not labeled as normal over threshold fixations. These severity alert signals indicate the user's proneness to possible threats. Further, the nutritional deficiency calculated is compared with the existential deficiency factor. These are followed by several cases where, if the user is predicted to have no deficiency, the message is displayed accordingly. However, if no previous deficiency was present and the new input indicates one, a nutrient deficiency warning is triggered. Based on age and recommended intake, the algorithm estimates how much more protein, iron, or omega-3 the user needs to fix the thresholds by suggesting a higher intake of lentils, eggs, leafy greens, or walnuts. If the deficiency is found to be decreased compared to before, it indicates the user is on track and no new diet is required. Visualizing these findings over a histogram leads to a better understanding of deficient cases.

In order to detect the deviation between actual and predicted values of hairfall rate against the hormonal fluctuation index, we use sinusoidal waves that reflect the consistency of the current condition. This algorithm creates a comparative graph over multiple users as subplots. These subplots are expressed as grids for comparative analysis, holding visual distinction of sine waves for existing matched data and cosine waves for new user input. This model also visualizes how the computed HFI of each user is compared to the existing HFI values in the dataset. Vertical lines that connect the existing and new HFI values using red and blue colors refer to the intensities of the HFI values, while the nodes are represented with black points and a horizontal dotted line in a distinct color, respectively, highlighting their position relative to the entire dataset.

Further, the role of the TFT function in our model is to forecast the future prediction of HFI over time. The graphical representation of the predictions displays the actual values as black-colored lines, which are constructed by learning from the historical data of recently observed HFI values. The predicted values are shown as blue lines, derived from past patterns and other internally learned attributes for future days, indicating the possible future deviations.

EXAMPLE:


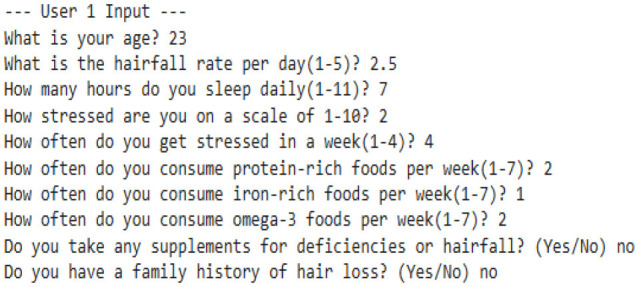



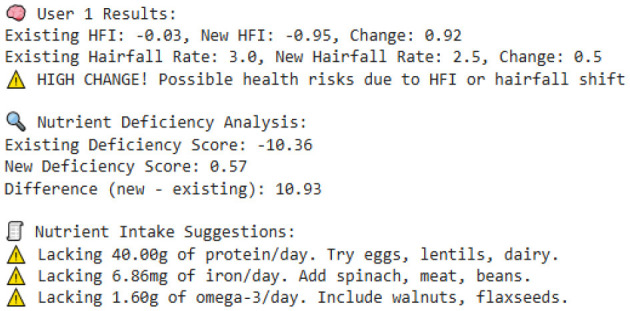



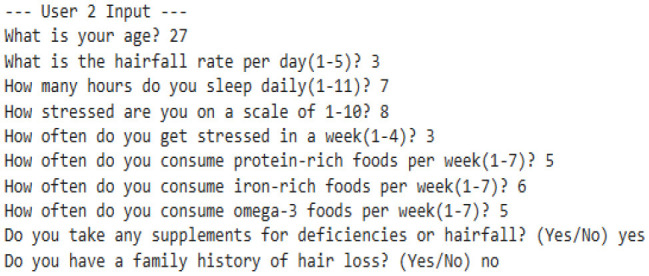



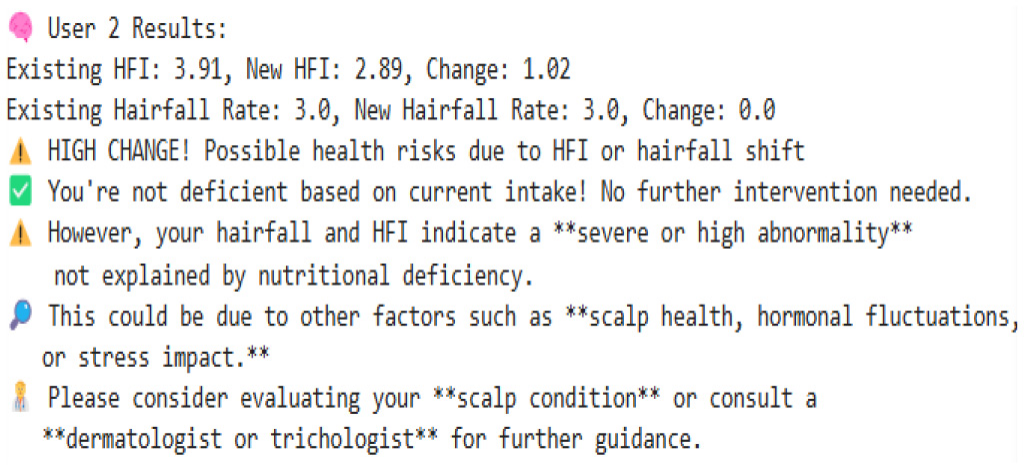



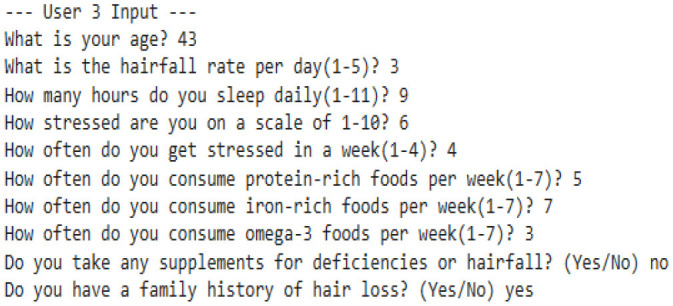



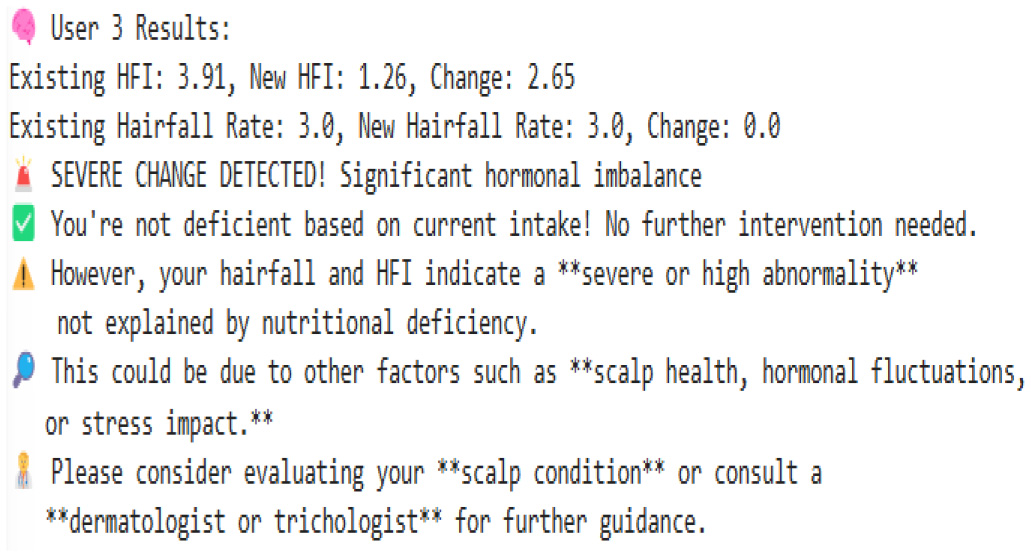



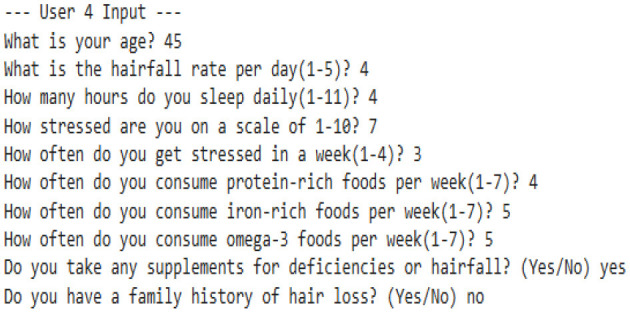



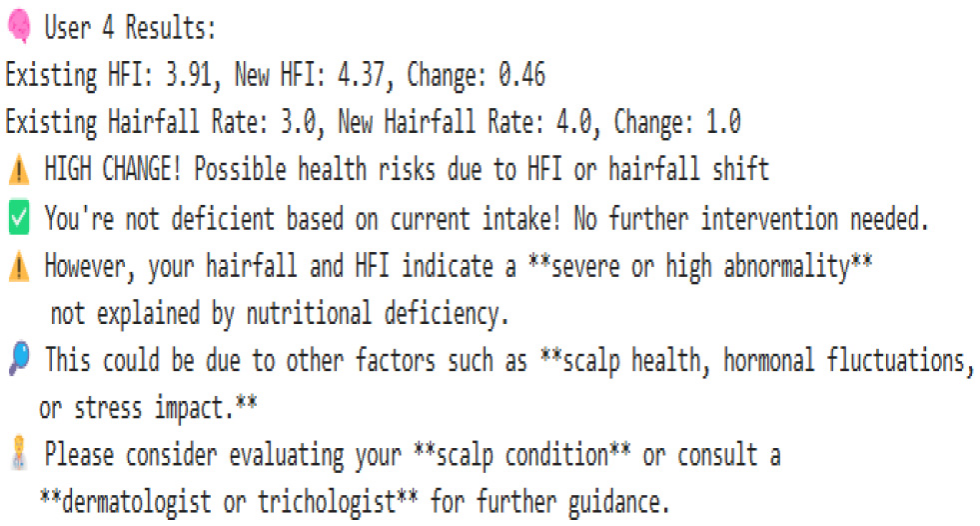


**Figure 8:**
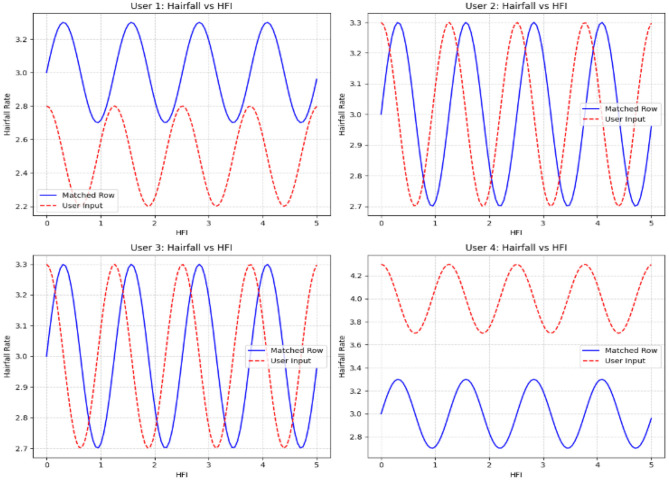
The subplots reveal the deviation of the Hairfall rate against HFI values for multi-user input cases.

Figure 8 displays the fluctuations of actual against predicted values, indicating the intensity over a range of deviations. Users 1 and 4 experience high deviation alerting threats, whereas users 2 and 3 experience almost no deviation, indicating less severity. This representation helps users understand trends in their hair loss, leading to personalized healthcare.

**Figure 9:**
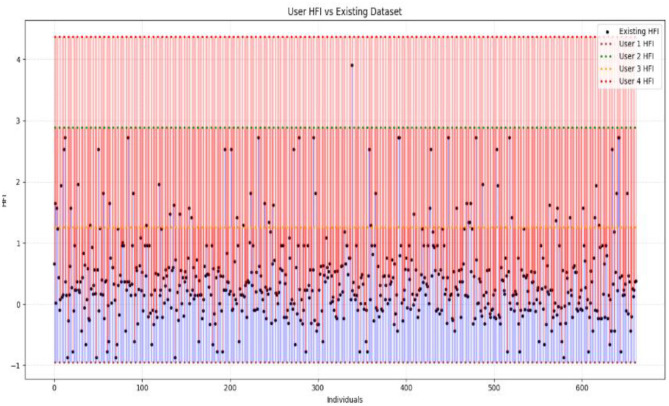
This graph shows the position of user HFI values in comparison to pre-existing HFI values.

The positioning of the user's HFI values over the clustered HFI values depicted in Figure 9 highlights the underlying categories by classifying the data into high and low. This enables us to find out if the value is a fit for existing data.

**Figure 10:**
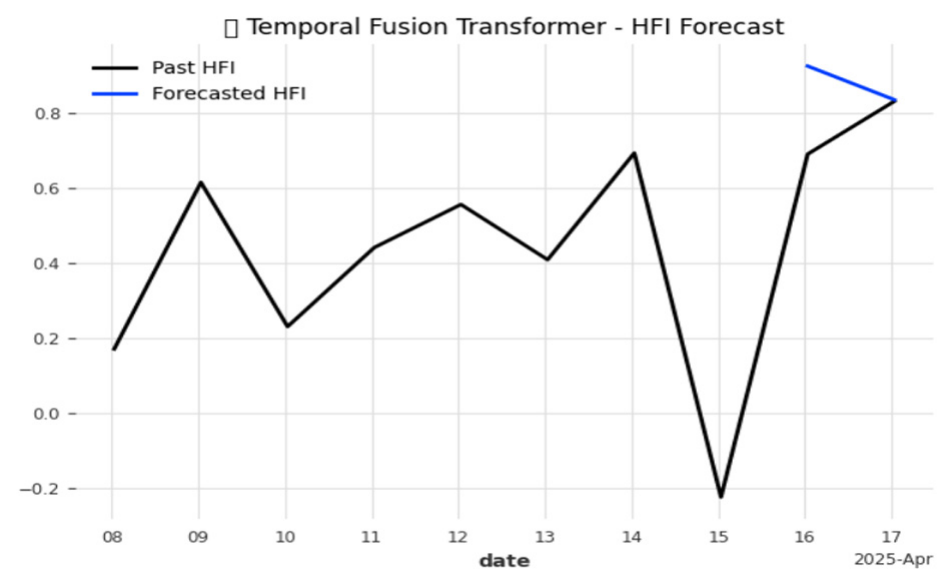
Temporal Fusion Transformer (TFT) forecast of the Hormonal Fluctuation Index.

The TFT model shown in Figure 10 predicts future HFI by learning from past patterns and relevant attributes such as nutrition, sleep, and stress. When this function is called, it fetches the pre-existing data for a particular user and uses time-based patterns to indicate future risks. This early warning demonstrates that the TFT model provides a time-aware, interpretable forecast, which is more advanced compared to other simple models.

Figure 10 displays how the Temporal Fusion Transformer (TFT) efficiently captures local changes in the Hormonal Fluctuation Index (HFI) and produces precise short-term predictions, such as identifying abnormal hair shedding patterns beforehand and facilitating the administration of suitable treatments.

### 8.2 Case study

#### 8.2.1 Case 1: moderate HFI deviation with lower hairfall rate

The system identifies a moderate deviation where the hormonal fluctuation is shifted from −0.27 to −0.80, and the hairfall rate dropped from 4.0 to 3.0. This case forecasts the possibility of a noticeable change in HFI (ΔHFI > 0.5), which previously indicated that there is a probability that the user could experience some threat in the future. Although the hairfall rate does not exceed the severe threshold, it has a considerable shift, which triggers a moderate anomaly alert. This enables the user to monitor and manage their health effectively. However, no dietary supplements are suggested, as the user is not nutritionally deficient. It also suggests that the user check for other factors or consult a dermatologist.

#### 8.2.2 Case 2: severe HFI and hairfall rate drop suggesting hormonal imbalance

This case exhibits a severe anomaly with a high HFI drop from −0.27 to −0.36, where the hairfall rate reduces from 4 to 2. This use case represents a high-risk scenario where both HFI and hairfall rate deviate significantly, exceeding the severe anomaly threshold (ΔHFI > 1.0 and ΔRate = 2), indicating a critical hormonal imbalance and threats that require medical attention. Nutritional health is not a concern, as current intake is sufficient, which rules out dietary deficiency. Even though the user is not deficient, there is a severe change in hormonal fluctuations; it is better to refer to a dermatologist immediately to check for other conditions and ensure that hormonal fluctuations are healthy enough to handle the hairfall rate drop.

#### 8.2.3 Case 3: high risk with nutrient deficiencies

The model detects a significant decrease in hormonal fluctuation of 0.64 units when compared to the existing value (ΔHFI = 0.64), indicating the possibility of threats in the future. This result is also combined with the notable drop in hair fall (ΔRate = 1.0), leading to early-stage detection of physiological disruption or threat conditions. Further, nutritional assessments detect a drastic shift in deficiency score from the preceding value. This symbolic shift from −9.05 to 0.43 units indicates the emergence of nutritional gaps, reflecting a daily shortage of 40 g of protein, 5.71 mg of iron, and 1.6 g of omega-3 fatty acids. Such deficiencies may directly contribute to declining health factors, including hairfall rates and hormonal fluctuations. As a result, the model suggests that supplementary diets—such as eggs, lentils, and dairy products for protein; spinach, meat, and beans for iron; and walnuts and flaxseeds for omega-3 intake—can help address these deficiencies. This case also leaves us with a histogram that effectively compares nutrient intake ratios over time, highlighting the algorithm's ability to detect underlying causes of HFI and hairfall anomalies while providing actionable dietary plans for intervention.

#### 8.2.4 Case 4: severe hormonal disruption with nutritional deficits

The extreme fluctuation in HFI by over 1.80 units from the existing value triggers the flag. However, the hairfall rate has decreased by 1 unit. This case shows a sudden drop within a certain period of time, detecting an anomaly in the series. This drastic change is a critical indicator of physiological instability. However, the user is indeed nutrient deficient, where the score changes from −9.79 to 0.14, which shows hidden nutritional insufficiencies, and our algorithm reports the deficits that need to be tackled, suggesting appropriate dietary plans to be followed to address 19.43 g of protein, 4.57 mg of iron, and 1.80 g of omega-3 deficiency. This model's strength lies not only in detecting severe hormonal anomalies but also in delivering personalized nutritional strategies to support holistic recovery and balance.

#### 8.2.5 Case 5: normal fluctuations with no deficiencies

This scenario illustrates a scenario where there is no deficiency and the person is perfectly fine. The mild variation in the Hormonal Fluctuation Index, by 0.36 units, and no change in hairfall rate demonstrate the stabilized health indicators of the user. Further, the nutrient deficiency test is also passed, confirming that the user's dietary plan is sufficient, and it is better to continue with that. This case demonstrates the model's ability to identify stable users and suppress false alarms, thereby supporting its accuracy and credibility in distinguishing between normal and abnormal states.

#### 8.2.6 Case 6: detecting nutrient deficiencies despite normal hormonal fluctuations

This case represents mild yet stable changes, with a small positive shift in hormonal fluctuation by 0.44 and a slight decrease in the hairfall rate by 0.5 units. This indicates that the user is out of threat and stabilized, lying within the range with no abnormalities. It flags the normal fluctuations without raising errors and performs deficit analysis. It reveals that the person, despite having stabilized fluctuations, still has some deficiencies that may affect them in the future. The scores have significantly risen from −10.36 to 0.43, indicating issues in dietary intake and suggesting the user take 48 g of protein, 5.7 mg of iron, and 1.37 g of omega-3 as per the deficits, which are crucial for hair growth and scalp health. This highlights that the model efficiently identifies nutritional gaps, allowing for prevention even when primary indicators appear to be normal.

## 9 Improvisation

Traditional hairfall detection methods involve biological testing, which includes analyzing blood samples to identify deficiencies or hormonal imbalances. Over time, they have been replaced with machine learning algorithms, such as CNN, SVM, and KNN, for image recognition and interpretation to detect anomalies. Although effective in certain cases, these models require high-resolution images, large datasets, and sophisticated image recognition pipelines, which make them complicated, resource-intensive, and lacking in scientific or numeric interpretability. Furthermore, many individuals are unaware or lack the necessary technical knowledge to consistently capture and upload images documenting their hairfall condition. Our method addresses all these constraints by gathering user input data, which is collected based on simple and understandable questions that users can easily answer, and building significant attributes for deeper analysis. The key improvisation is the integration of time-centric data, allowing us to capture abnormal deviations in hairfall rate over time, which is the core innovation of this project. By employing numerical data rather than images, our model is more scalable, interpretable, and diverse. It also involves several contributing factors, such as nutrient deficiencies, exposure to pollution, and stress levels, enabling a comprehensive and intelligent analysis of lifestyle factors.

## 10 Results and discussions

This article describes the work of creating a machine learning methodology using LSTM, Random Forest, Temporal Fusion Transformer (TFT), and ARIMAX models to predict the degree of hairfall from time-series data. Additionally, a comprehensive dataset comprising five structured data categories—stress level, sleep cycles, dietary intake, scalp health, and hormone imbalance—was utilized for model training and validation. Ultimately, only the Temporal Fusion Transformer (TFT) model was confirmed as the most promising one, performing on all major metrics, yielding the best possible results with 97.52% accuracy, 97.25% recall, and an R^2^ score of 0.98, and the smallest possible MSE of 0.03. The way this investigation stands out is by presenting an innovative perspective based on the Hormonal Fluctuation Index (HFI) tool, which harmonizes the human factor with the computer engine to identify hairfall trends on a micro or macro level. Simultaneously, the model uses multiple components related to the HFI in a real-time manner and maintains constant feedback to predict human health changes. This prevented the hairfall if it was not suddenly too high as predicted. The model also advises the subject on his/her dietary needs (if there is any nutrient deficit), suggesting that protein, iron, and omega-3 are good sources consistent with the user's health profile. In this way, the model becomes an accurate identifier and preventive care provider through personalized recommendations, as well as an early alert system. The coupling of health data with hair forecast monitoring represents an innovative approach to health management through proactive and predictive measures. The forecasts generated by the model developed here encompass a diverse range of hormone changes and nutritional intake, providing a combination of molecular understanding and actionable information that supports the transition from reactive hair loss prediction to proactive management of the situation. This technique flawlessly addresses the main question of detecting hair loss as early as possible and provides suitable, timely recommendations for lifestyle or dietary changes tailored to individual characteristics. Moreover, the transparency aspects of the TFT model—such as variable selection importance scores and time-dependent attention weight distributions—provide clear indications about what kind of lifestyle, dietary, and stress factors have the greatest impact on the model's forecast. In this case, trust in the model is improved, allowing users to take personalized and practical actions. A new direction that this machine can take is connecting it with medical data, which can carry enough information for the machine to give clinical advice. This will also operate as an effective spare part for the clinicians regarding shared decision-making in the early stages.

### 10.1 Comparative evaluation with existing literature

Our methodology differs from most research on predicting hair loss, which primarily relies on high-resolution scalp or hair images with CNN-based classifiers or on clinical datasets containing biochemical markers and dermatological assessments. Rather, it is based on self-reported, multivariate time-series data that encompasses lifestyle and nutritional factors, thereby eliminating the need for specialized imaging equipment or invasive clinical measurements. Owing to the fundamental differences in input modality, feature composition, and data collection protocols, simply recreating prior image-based or clinical models on our dataset will not result in a fair or meaningful performance comparison. Therefore, we provide a qualitative comparison with representative CNN-based image classifiers and ML-based health prediction models, highlighting their differences in data requirements, computational complexity, and practicality in real-world, non-clinical settings. Our baseline models—LSTM, GRU, TCN, Random Forest, and ARIMAX—were chosen for their suitability to temporal, multivariate forecasting tasks and, therefore, they enable a fair and robust comparison within the same data domain. This study marks the debut of the Temporal Fusion Transformer (TFT) application in hairfall anomaly detection with lifestyle-linked time-series data. The TFT method offers several advantages, including high interpretability, low deployment costs, and a minimal data acquisition burden; therefore, it is a viable and scalable option compared to image-based diagnostic methods.

## 11 Use cases

The outlined system is believed to have a significant impact. It utilizes TFT, a powerful machine learning model that helps the system identify unusual patterns, providing accurate results and offering useful suggestions that can lead to early detection and improved health outcomes. One of the primary applications of this model is in personalized hair health monitoring. Through this, individuals can track hairfall patterns on a daily or weekly basis, allowing the system to detect anomalies. This enables timely alerts to proclaim possible underlying health risks, such as hormonal imbalances, stress, or poor dietary habits. In the field of clinical and dermatological care, this framework can be employed as a supportive tool for diagnosis. Instead of depending solely on visual inspection or high-cost image processing methods, clinicians can use derived indices, such as the Hormonal Fluctuation Index (HFI), Nutrient Deficiency Score, and Scalp Health Score, to understand the root causes of hairfall. These derived metrics offer transparency and reliability, making them ideal for use in dermatological consultations and for treatment planning.

Another key application lies in nutritional and lifestyle advisory systems. The model detects dietary deficiencies related to iron, protein, and omega intake and suggests food intake recommendations. By integrating biological and behavioral data with nutritional science, the system helps promote wellness, positioning the framework as a valuable extension to preventive healthcare. Moreover, the system serves as an early indicator of systemic health disorders. Since an abnormal hairfall rate often reflects physiological or psychological issues, such as anemia or chronic stress, it can serve as a predictive interface between personal health tracking and medical diagnostics.

Upon closer examination, this research can make significant contributions to public health and disease studies. With organized data, researchers can study hair loss patterns in different age groups, regions, and environments. These findings can support public health policies, research on how climate affects health, and awareness programs about health issues caused by poor nutrition and stress. Additionally, this model adds value to the haircare and pharmaceutical industries by providing insights into consumer behavior. Brands can use real-time user data to understand the performance and customer acceptance of the products and thus create targeted marketing tactics. This consumer-centric approach, driven by data, not only aids in R&D but also delivers an extra spark to clients through individualized service offerings. To sum up, the suggested model serves not only as a unique method for self-health tracking but also as a multifunctional instrument for purposes ranging from medical diagnoses to business product inventions.

## 12 Future scope

Future improvements to the hairfall prediction system can include seasonal factors, as changes in humidity, pollution, temperature, and UV exposure significantly impact scalp and hair health. For example, higher hairfall is frequently observed in monsoon and autumn and is usually associated with fungal infections of the scalp or a breakdown in the natural cycle of hair growth. By integrating such seasonal dynamics into the model, predictions could be enhanced not just for personal health metrics but also for external environmental stressors, thereby improving the adaptability and relevance of the system. To enhance the model by integrating medical profiles such as symptoms related to anemia, chronic stress, thyroid dysfunction, and PCOS, which enables the model to correlate specific hairfall conditions with anomalies. This would improve the model's medical relevance and improve its predictive capability.

Furthermore, incorporating longitudinal data from wearable health trackers can provide real-time insights by collecting lifestyle metrics, such as heart rate variability, blood pressure levels, and oxygen saturation, which serve as stress markers, thereby enhancing predictive accuracy. Additionally, deploying this model in a mobile health application can enable users to track their hair health trends, receive alerts, and receive personalized recommendations based on their changing risk profile. These enhancements not only improve the accuracy of prediction but also affirm the model's utility in preventive healthcare and personalized wellness management.

## 13 Conclusion

This research aimed to develop an interpretable and active framework for predicting hairfall rate by emphasizing hormonal fluctuations as a key determinant. Traditional models, which rely on visual data, have been replaced with a structured, interpretable approach that utilizes physiological, behavioral, and lifestyle attributes. By introducing derived indices, such as the Hormonal Fluctuation Index (HFI), and implementing several machine learning models, our analysis reveals TFT as the most robust model, based on performance metrics, as mentioned in the performance analysis, and for accurately predicting anomalies in time-series data. The system that we are suggesting identifies strange hair loss patterns and issues, personal alarms, and diet tips. It is effective in everyday activity and provides understandable, health-related results. For instance, it can be further advanced by incorporating climatic conditions and medical symptoms, which will assist in the early identification and prevention of the issue.

## Data Availability

The raw data supporting the conclusions of this article will be made available by the authors, without undue reservation.
